# Novel stress granule-like structures are induced via a paracrine mechanism during viral infection

**DOI:** 10.1242/jcs.259194

**Published:** 2022-03-02

**Authors:** Valentina Iadevaia, James M. Burke, Lucy Eke, Carla Moller-Levet, Roy Parker, Nicolas Locker

**Affiliations:** 1 Faculty of Health and Medical Sciences, School of Biosciences and Medicine, University of Surrey, Guildford GU2 7HX, UK; 2 Department of Biochemistry, University of Colorado, Boulder, CO 80303, USA; 3 Howard Hughes Medical Institute, University of Colorado, Boulder, CO 80303, USA

**Keywords:** Virus, Stress granule, G3BP1

## Abstract

To rapidly adapt to stresses such as infections, cells have evolved several mechanisms, which include the activation of stress response pathways and the innate immune response. These stress responses result in the rapid inhibition of translation and condensation of stalled mRNAs with RNA-binding proteins and signalling components into cytoplasmic biocondensates called stress granules (SGs). Increasing evidence suggests that SGs contribute to antiviral defence, and thus viruses need to evade these responses to propagate. We previously showed that feline calicivirus (FCV) impairs SG assembly by cleaving the scaffolding protein G3BP1. We also observed that uninfected bystander cells assembled G3BP1-positive granules, suggesting a paracrine response triggered by infection. We now present evidence that virus-free supernatant generated from infected cells can induce the formation of SG-like foci, which we name paracrine granules. They are linked to antiviral activity and exhibit specific kinetics of assembly-disassembly, and protein and RNA composition that are different from canonical SGs. We propose that this paracrine induction reflects a novel cellular defence mechanism to limit viral propagation and promote stress responses in bystander cells.

## Abstract (precis)

**Summary:** We describe novel RNP granules induced by viruses that exhibit a distinct protein and RNA composition from stress granules, and are associated with translational shut-off and antiviral activities.

## INTRODUCTION

Controlling the localisation and function of macromolecules is central to cell biology and can be achieved by surrounding them with lipid membranes in organelles such as the nucleus, lysosomes or mitochondria. Membraneless compartments, known as biocondensates or membraneless organelles, are increasingly recognised as an alternative way to organise cellular components. They are maintained through a combination of protein-protein, protein-RNA and RNA-RNA interactions, and their dynamic formation generates high local concentrations of RNA and protein (Tauber et al., 2020). Because of this remarkable molecular plasticity, biocondensates provide an ideal platform for the regulation of cellular fundamental processes, such as mRNA metabolism or intracellular signalling, and are utilised by cells to rapidly adjust and rewire regulatory networks in response to various physiological and pathological triggers (Yoo et al., 2019).

Stress granules (SGs) are among the most characterised cytoplasmic biocondensates (Corbet and Parker, 2019; Hofmann et al., 2020). They are important for the organisation of cellular content, capturing mRNAs and proteins during stresses including oxidative stress, heat shock, viral infection, proteasomal inhibition, ER stress and UV irradiation, among others (Hofmann et al., 2020). The general inhibition of protein synthesis following stress, usually triggered by phosphorylation of the eukaryotic translation initiation factor 2α (eIF2α), results in the dissociation of mRNAs from polysomes and their accumulation in ribonucleoprotein (RNP) complexes (Hofmann et al., 2020). This increased concentration of cytoplasmic RNPs and their binding by aggregation prone RNA-binding proteins (RBPs), such as Ras-GTPase activating SH3 domain-binding protein 1 (G3BP1) and T cell internal antigen-1 (TIA-1), results in the recruitment of multiple proteins characterised by the presence of low sequence complexity, intrinsically disordered regions in their structures. These mediate clustering and fusion events driven by multivalent interactions between their protein and RNA components, with G3BP1 acting as a key node for promoting RNA-protein, protein-protein and RNA-RNA interactions, ultimately resulting in liquid-liquid phase separation (LLPS) and SG formation (Corbet and Parker, 2019; Wang et al., 2018). SGs are highly dynamic, rapidly assembling to sequester the bulk content of cytoplasmic mRNAs, and dissolving upon stress resolution to release stored mRNAs for future translation (Matheny et al., 2019; Namkoong et al., 2018).

In addition to a general role during the inhibition of translation, SGs can also be stress-selective in composition and function (Aulas et al., 2017). Recent studies have proposed they may adopt non-homogeneous structures with variable compositions dependent on the stress and have proposed classifying SGs into three types (Hofmann et al., 2020). Type I canonical SGs form via an eIF2α-dependent pathway, whereas type II SGs assemble following eIF2α-independent inhibition of translation. In contrast, type III SGs lack eIFs and are associated with cellular death (Reineke and Neilson, 2019). This suggests that compositionally heterogeneous SGs support specialized functions promoting survival or pro-death outcomes. By sequestering specific proteins, SGs alter the composition and concentration of cytoplasmic proteins, which in turn can change the course of biochemical reactions and signalling cascades in the cytosol (Riggs et al., 2020). Moreover, mutations impacting SG clearance or dysregulating LLPS can lead to persistent or aberrant SGs, which are increasingly associated with neuropathology, in particular amyotrophic lateral sclerosis (ALS) and related diseases (Wolozin and Ivanov, 2019). Many SG proteins are also aberrantly expressed in tumours, and SGs are exploited by cancer cells to adapt to the adverse conditions of the tumour microenvironment (Anderson et al., 2015).

Importantly, SGs are at a crossroads between intracellular signalling, antiviral responses and translation control through concentrating key signalling and cytoplasmic sensors or effectors of innate immunity (Eiermann et al., 2020). Recent work has proposed that SGs exert antiviral activities by providing a platform for antiviral signalling (Eiermann et al., 2020). A well-known antiviral response is the induction of type I interferons (IFNs). During viral replication, double-stranded RNA replication intermediates can be recognized by cytoplasmic sensors such as RIG-I-like receptors (RLRs) or the eIF2α kinase PKR (EIF2AK2) to amplify the IFN response and create an antiviral state (Eiermann et al., 2020; Mateju and Chao, 2021). Multiple IFN signalling molecules, including PKR, MDA5 (also known as IFIH1), RIG-I (also known as DDX58) and TRAF2, can be recruited to SGs, and this localization has been suggested to regulate their activity (Eiermann et al., 2020; Mateju and Chao, 2021). Furthermore, SGs or specific antiviral SGs (avSGs) have been proposed to play a role in antiviral signalling, as key signalling proteins including MDA5 and PKR are known to localise to SGs and SG formation is involved in PKR activation (Eiermann et al., 2020; Mateju and Chao, 2021). Because of this proposed role in antiviral signalling and impact on cellular protein synthesis which they rely on, many viruses have evolved strategies to antagonize or exploit SGs, for example by cleaving or repurposing SG-nucleating proteins during infection or impairing the eIF2α sensing pathway (Gaete-Argel et al., 2019). Among these, G3BP1 is a prime target and it is proteolytically cleaved by enterovirus and calicivirus proteases, sequestered by the alphavirus nsp3 protein or repurposed during dengue and vaccinia virus infection (Gaete-Argel et al., 2019). Interestingly, even closely related viruses use different strategies to counteract SGs. Indeed, we previously demonstrated that although feline calicivirus (FCV) disrupts the assembly of SGs by inducing G3BP1 cleavage through its 3C-like protease, infection with the related murine norovirus (MNV) has no impact on G3BP1 integrity (Brocard et al., 2020; Humoud et al., 2016). Instead, viral proteins interact with G3BP1, resulting in its relocalization to replication complexes and in the reshaping of its interactions with cellular partners, repurposing G3BP1 as a viral translational enhancer (Hosmillo et al., 2019).

Previous analysis of FCV infection revealed that some uninfected cells near infected cells displayed G3BP1 foci. Herein, we demonstrate that infected cells communicate to nearby bystander cells, resulting in the assembly of SG-like foci we name paracrine granules (PGs). Importantly, although PGs and arsenite-induced SGs share many components, such as mRNAs of similar functional families and some resident proteins, PGs exhibit specific features. First, their assembly-disassembly patterns are different: PG assembly can occur in the absence of the SG scaffold G3BP1 and their disassembly is faster. Second, despite being associated with translational shut-off, their assembly is insensitive to cycloheximide and blocking of mRNAs onto polysomes. Finally, PG induction impairs viral replication, suggesting a role in preventing or reducing viral propagation. Therefore, we propose a model in which the assembly of PGs is a pro-survival event resulting from stress signals sent from infected cells to the nearby environment.

## RESULTS

### FCV infection induces granule formation in a paracrine manner

**Fig. 1.**
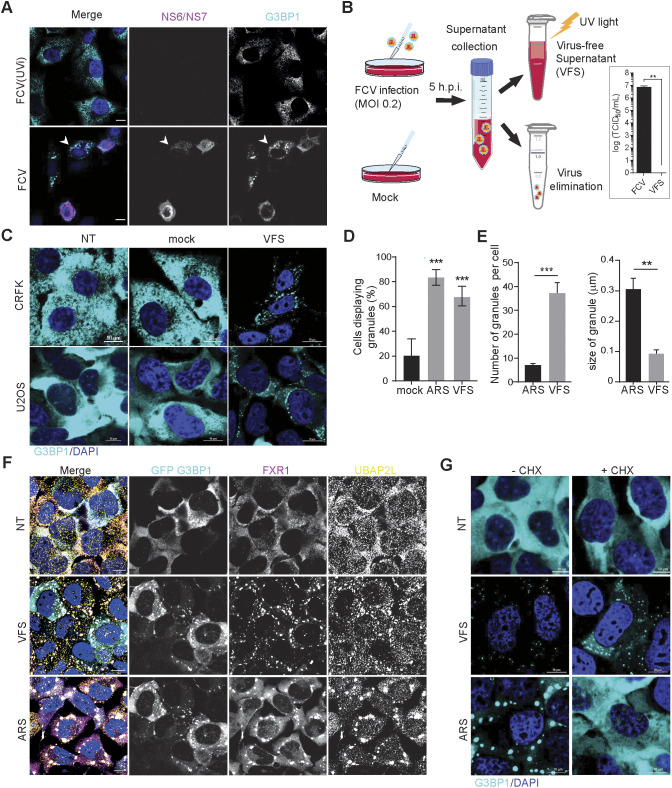
**FCV infection results in the formation of paracrine granules.** (A) Confocal microscopy analysis of CRFK cells infected with FCV (MOI 0.2) or UV-inactivated FCV [FCV(UVi)] for 5 h. Samples were stained for the SG marker G3BP1 (cyan) and infected cells detected by immunostaining against FCV NS6/7 (magenta), and the nuclei were stained with DAPI (blue). Scale bars: 20 μm. White arrowheads indicate G3BP1 foci. (B) Schematic representation of the VFS preparation procedure. Detection of FCV replication by TCID_50_ assay following VFS treatment or infection of CRFK cells with FCV at MOI of 2. Results are mean±s.e.m. (*n*=3). Statistical significance is shown above the bars. **P<0.01 (one-tailed unpaired *t*-test). (C) CRFK or U2OS GFP-G3BP1 cells were stimulated for 1 h with VFS from a mock or FCV infection and the distribution of G3BP1 analysed by confocal microscopy using detection of endogenous G3BP1 (cyan) in CRFK, or GFP (cyan) in U2OS cells. Non-treated (NT) cells were used as controls. Nuclei were stained with DAPI (blue). Scale bars: 10 μm. (D) Bar plot (*n*=3) of the percentage of U2OS cells displaying G3BP1 foci, mean±s.d. for 100 G3BP1-positive cells analysed across at least 10 acquisitions. ARS, 0.5 mM sodium arsenite treatment. Statistical significance shown above the bars, ***P<0.001 (one-way ANOVA). (E) Bar plot (*n*=3) of the number of G3BP1 granules per cells displaying G3BP1 foci and the average granule size, mean±s.d. for 100 G3BP1-positive cells analysed across at least 10 acquisitions. Statistical significance shown above the bars, ***P<0.001, **P<0.01 (one-way ANOVA). (F) U2OS GFP-G3BP1 cells were stimulated for 1 h with VFS or 0.5 mM sodium arsenite (ARS) prior to fixation and the formation of PGs or SGs analysed by confocal microscopy. Non-treated (NT) cells were used as controls. Cells were stained with GFP (cyan), FXR1 (magenta) and UBAP2L (gold) as PG or SG markers. Nuclei were stained with DAPI (blue). Scale bars: 10 μm. (G) U2OS GFP-G3BP1 cells were pretreated with ARS or VFS and for forced SG disassembly, treated with 10 μg/ml of CHX for the final 30 min (+CHX). The presence of G3BP1 granules was assessed as in D. Scale bars: 10 μm. Images in A,C,F and G are representative of three experiments.

We previously reported that FCV infection impairs SG accumulation in infected Crandell–Rees feline kidney (CRFK) cells through NS6-mediated proteolytic cleavage of the SG scaffolding protein G3BP1 (Humoud et al., 2016). Intriguingly, this also revealed that a small fraction of uninfected cells assembles SG-like foci during infection at low multiplicity of infection (Fig. 1A). This suggests a possible paracrine induction of SG-like foci assembly that could contribute to impairing viral replication or propagation in uninfected cells. To test this hypothesis, we generated virus-free supernatant (VFS) from FCV-infected or mock-treated cells (Fig. 1B). Briefly, the cell culture supernatant was collected, and the viral particles were precipitated using PEG3350 and NaCl and removed via ultracentrifugation and UV inactivation, as shown in Fig. 1B. The VFS generated was assayed for the presence of infectious viral particles by measuring viral titres after incubation with CRFK cells for up to 72 h using 50% tissue culture infectious dose (TCID_50_) assays, which confirmed the removal of infectious particles. We then tested the ability of the VFS to induce SG assembly in either CRFK or U2OS cells expressing GFP-G3BP1. Cells were stimulated for 1 h with VFS or arsenite, which induces SG by activating the eIF2α kinase HRI (EIF2AK1) (Taniuchi et al., 2016), fixed and labelled with an anti-G3BP1 antibody to detect SG assembly. As expected, arsenite treatment resulted in the assembly of SGs, reflected by the accumulation of G3BP1 into cytoplasmic foci (Fig. 1C). Similarly, treatment of both CRFK and U2OS cells with VFS resulted in the formation of G3BP1 foci in the cytoplasm (Fig. 1C). Detailed analysis in U2OS cells revealed that arsenite and VFS treatments resulted in the formation of G3BP1 foci in 85% and 70% of cells, respectively (Fig. 1D). Interestingly, further analysis also revealed that the average size of foci induced by VFS was significantly smaller than the size of arsenite-induced SGs, and that the number of foci per cell was significantly higher in the VFS-treated compared to the arsenite-treated cells (Fig. 1E), suggesting a different nature of foci induced. We confirmed these results by assessing the presence of other SG resident proteins, the RBPs FXR1 and UBAP2L. Following stimulation with arsenite or VFS, FXR1 and UBAP2L colocalised with G3BP1 in U2OS GFP-G3BP1 cells, and analyses further supported that VFS triggered the formation of smaller and more abundant cytoplasmic foci (Fig. 1F; Fig. S1A,B). Thereafter, the VFS-induced G3BP1 foci were named paracrine granules (PGs).

To further characterize the features of PGs, their susceptibility to cycloheximide (CHX) treatment was determined. The assembly of canonical SGs is dependent on the shuttling of mRNAs dissociating from polysomes into SGs. By binding to ribosomes and preventing mRNA release, CHX inhibits SG formation (Kedersha et al., 2000). U2OS GFP-G3BP1 cells were pretreated with CHX and then treated with either arsenite or VFS. As expected, CHX impaired the assembly of canonical SGs; however it was unable to block PG formation following VFS treatment, and PGs appeared larger following CHX treatment (Fig. 1G). This strongly suggests that PGs form in response to stimuli different from canonical SGs.

Next, we examined the kinetics of assembly-disassembly of PGs compared to SGs using time-lapse confocal microscopy (Fig. S1). First, G3BP1 foci formation was recorded by collecting images every 10 min for 180 min, and the disassembly of these foci upon stressor removal was recorded in a similar manner. As shown in Fig. S1C, PGs formed faster than the SGs, with 10 min stimulation with VFS sufficient to induce PG assembly, whereas canonical SGs required a longer time, between 30 and 40 min of arsenite treatment, in order to form completely. More strikingly, following foci disassembly, recovery assays revealed that although canonical SGs required up to 3 h to disassemble, PGs completely disappeared within 20 min of stress removal (Fig. S1D), potentially reflecting a more dynamic nature of these condensates. To test this further, the internal mobility of GFP-G3BP1 was measured using fluorescence recovery after photobleaching (FRAP) and cells were treated with either VFS or arsenite. Surprisingly, we could not detect any differences in the recovery of GFP-G3BP1 fluorescence after photobleaching, and thus in G3BP1 mobility between the two conditions (Fig. S1E). Overall, these data suggest that VFS from infected cells induce the formation of PGs that display specific features of assembly and disassembly dynamics, different from canonical SGs.

### G3BP1 is not essential for PGs assembly

**Fig. 2.**
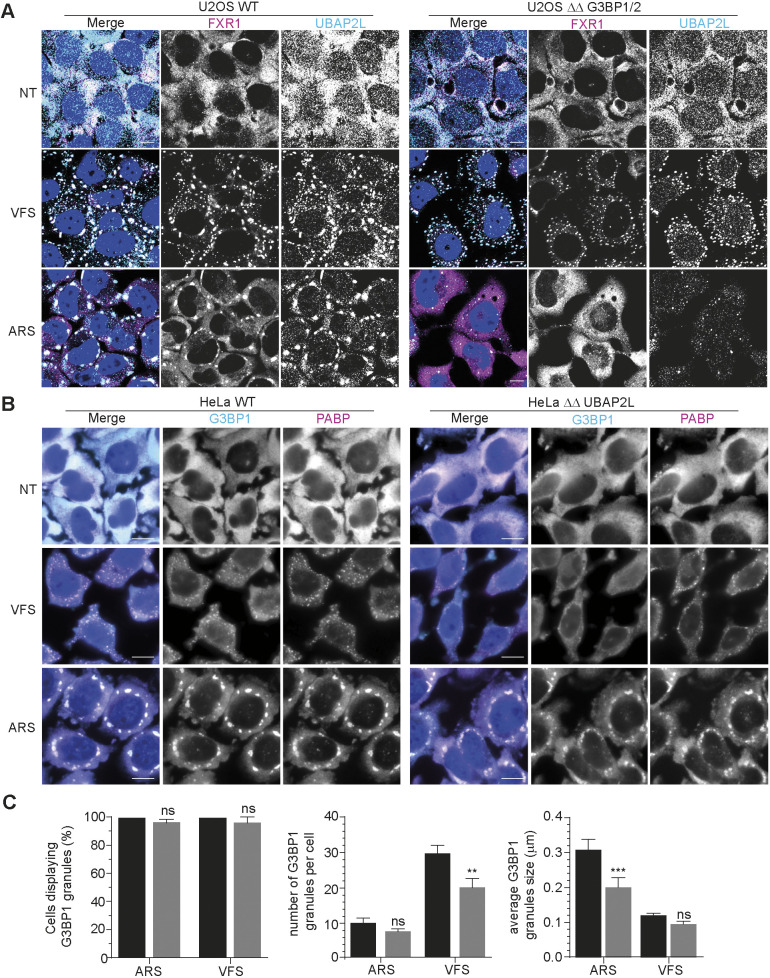
**G3BP1 is not an essential for PG assembly.** (A) Wild-type (WT) or ΔΔ G3BP1/2 U2OS cells were stimulated for 1 h with VFS or 0.5 mM sodium arsenite (ARS) prior to fixation and the formation of PGs or SGs analysed by confocal microscopy. Non-treated (NT) cells were used as controls. Cells were stained with FXR1 (magenta) and UBAP2L (cyan) as PG or SG markers. Nuclei were stained with DAPI (blue). Scale bars: 10 μm. (B) Wild-type (WT) or ΔΔ UBAP2L HeLa cells were stimulated as in A prior to fixation and the formation of PGs or SGs analysed by confocal microscopy. Non-treated (NT) cells were used as controls. Cells were stained with G3BP1 (cyan) and PABP1 (PABP, magenta) as PG or SG markers. Nuclei were stained with DAPI (blue). Scale bars: 10 μm. (C) Bar plot (*n*=3) of the average number of WT or ΔΔ UBAP2L HeLa cells with G3BP1 granules, the number of G3BP1 granules per cells displaying G3BP1 foci and the average granule size, mean±s.d. for 100 cells analysed across at least 10 acquisitions. Black, WT; grey, ΔΔ UBAP2L. Statistical significance shown above the bars. ***P<0.001; **P<0.01; ns, not significant (two-way ANOVA).

With strong indication that PGs and SGs share common features but exhibit fundamental differences, we next investigated the importance of G3BP1 for PG assembly. G3BP1 is central and acts as a molecular switch for SG assembly (Yang et al., 2020). Following translation inhibition, the increase in cytoplasmic mRNAs facilitates the clustering of G3BP1 through protein-RNA interactions into networked condensates. These condensates recruit additional client proteins that promote LLPS and SG maturation. Importantly, during SG nucleation, cores can also assemble around the RBP UBAP2L, even in absence of G3BP1 (Cirillo et al., 2020). First, we investigated the requirement for G3BP1 during PG assembly. To this end, U2OS wild-type (WT) or G3BP1 and G3BP2 double knockout (ΔΔ G3BP1/2) cells were treated with VFS or arsenite and stained for the PG and SG core markers UBAP2L and FXR1. In untreated WT or ΔΔ G3BP1/2 cells, both FXR1 and UBAP2L remained diffused in the cytoplasm (Fig. 2A). In contrast, VFS treatment resulted in the assembly of PGs that contained both FXR1 and UBAP2L in either U2OS WT or ΔΔ G3BP1/2 cells (Fig. 2A). Next, given its role in driving the nucleation and maturation of SGs, we tested the requirement for UBAP2L during PG assembly. HeLa WT or UBAP2L knockout (ΔΔ UBAP2L) cells were treated with VFS or arsenite and stained for the PG and SG markers G3BP1 and PABP1 (encoded by *PABPC1*) (Fig. 2B). Although the absence of UBAP2L did not block the formation of PGs or SGs, it resulted in smaller SGs as proposed before and a lower number of PGs per cell (Fig. 2C) (Cirillo et al., 2020). Therefore, although not essential for PG assembly, we propose that UBAP2L contributes to PG maturation. Overall, these results further highlight the heterogenous nature of PGs whose assembly, unlike SGs, is not dependent on the presence of G3BP1.

### Proteomics analysis of isolated PG reveals a distinct composition from SGs

**Fig. 3.**
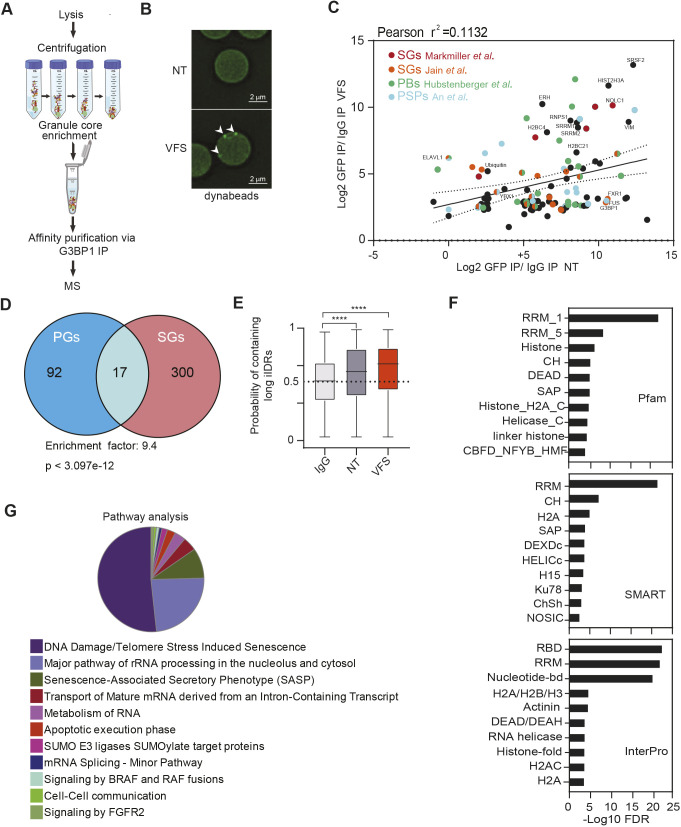
**Proteomic analysis reveals differences between VFS-induced PGs and ARS-induced SGs.** (A) Schematic representation of the granule isolation procedure. MS, mass spectrometry. (B) Protein A Dynabeads analysed by epifluoresence microscopy in non-treated (NT) or VFS-treated U2OS GFP-G3BP1 cells following PG isolation; bead-bound G3BP1 granules are indicated by white arrowheads. (C) Scatterplot of 110 proteins enriched in PGs over control (log_2_ transformed for each IP ratio relative to control IgG). Proteins identified as components of other biocondensates (SGs; PBs, P-bodies; PSPs, paraspeckles) are indicated in colour. The mean enrichment is shown by a solid line and dotted lines indicate the threshold for significance. (D) Venn diagram comparison of 110 proteins identified in PGs and 317 in SGs (from Jain et al., 2016). The representation factor shows the enrichment over the expected, and the *P*-value (two-way ANOVA) is based on the cumulative distribution function (CDF) of the hypergeometric distribution of the dataset over the mouse proteome. (E) Box-plots of the average score of the probability of a protein containing a long intrinsically disordered region determined using SLIDER in the GFP versus IgG IPs for VFS-treated or control (NT) cells. Lines mark the average values, boxes show the interquartile range of the probability values and whiskers indicate the range. The dotted line marks the average value for control IgG. Statistical significance shown above the bars, ****P<0.0001 (one-way ANOVA). (F) Bar plot of the most enriched protein domains in the PG discovery analysis using Pfam, SMART and InterPro. (G) GO pathway analysis of the 110 resident proteins isolated in PGs using Cytoscape.

Previous studies have suggested that in response to different stresses, SGs recruit specific resident proteins and that this SG heterogeneity might be important for their cellular functions (Reineke and Neilson, 2019; Youn et al., 2019). To explore this, we took advantage of an affinity-based SG isolation procedure we recently developed to characterise their proteome (Jain et al., 2016). To this end, U2OS GFP-G3BP1 cells were treated with VFS for 1 h to induce SG assembly. SG cores were then enriched by sequential centrifugation to generate a granule-enriched fraction and purified by immunoprecipitation (IP) using antibodies to GFP (to trap GFP-G3BP1) or IgG (as a control) followed by pull down with Protein A-conjugated Dynabeads as previously described and summarised in Fig. 3A (Brocard et al., 2020; Jain et al., 2016). Epifluorescence microscopy analysis then confirmed the isolation of SG cores on anti-GFP beads, while no SG cores could be detected in the control IgG IP (Fig. 3B). The background halo GFP staining detected in control conditions agrees with previous studies that reported that G3BP1-mediated protein-protein interactions pre-exist in the cytoplasm in the absence of stress but only coalesce into large granules upon stress (Brocard et al., 2020; Jain et al., 2016)*.* To characterize the identity of PG resident proteins, mass spectrometric analysis by liquid chromatography-tandem mass spectrometry (LC-MS/MS) was performed on proteins eluted from the beads and analysed using MaxQuant. A total of 667 proteins were detected with a false-discovery rate (FDR) of less than 5%, 191 proteins displayed at least two different peptides, and applying a filtering criterion of ≥1 log_2_-fold changes of immunoprecipitated proteins compared to the respective control IgG conditions, we finally identified 110 proteins enriched in the PGs (Table S1).

Next, we compared the PG protein composition with that of previously published arsenite-induced SGs (Jain et al., 2016; Markmiller et al., 2018) and other biocondensates such as P-bodies (PBs) (Hubstenberger et al., 2017) and paraspeckles (PSPs) (An et al., 2019) (Fig. 3C; Table S2). This highlighted that the composition of PGs is widely different from these other biocondensates. More specific comparison of the previously established arsenite-induced SG proteome in U2OS cells revealed that PGs share a number of components with canonical SGs, as shown in Fig. 3D and Table S3 (Jain et al., 2016), with 17 out of 110 PG proteins displaying a significant enrichment in SGs (946-fold enrichment, *P*<3.097×10^–12^). These include RBPs such as FXR1 and ELAVL1, and RNA helicases such as DDX1 and DDX21. We further confirmed the cellular distribution of some of these targets using immunofluorescence following treatment with either arsenite or VFS. The known SG residents ELAVL1, FXR1 and UBAP2L colocalised with G3BP1 both in PGs and SGs (Fig. S2). In contrast, HNRNPK, THRAP3 and RBMX colocalised with G3BP1 in PGs but not in arsenite-induced SGs, confirming the assembly of compositionally distinct foci (Fig. S3). Because canonical SGs condense translationally stalled RNPs released from polysomes, previous proteomics studies have identified the presence of translation initiation factors such as eIF3 subunits, eIF2, eIF4G, eIF4A, eIF4B and eIF4H, and small but not large ribosomal proteins (Jain et al., 2016; Markmiller et al., 2018). In contrast, PG resident proteins included both small and large ribosomal proteins (e.g*.* rpS14, rpL4, rpL6, rpL17, rpL19), but only eIF5b and none of the eIF3, eIF2 or eIF4F complex subunits, pointing to a fundamental difference in composition.

RNase L-bodies (RLBs) are small SG-like puncta that were previously shown to assemble independently from G3BP1, like PGs, in response to non-self sensing by RNase L (Burke et al., 2020). We therefore interrogated whether PGs and RLBs are distinct SG-like foci by comparing their protein composition. Despite a higher proportion of shared components, and a higher enrichment factor, with RLBs rather than SGs or other biocondensates, this revealed a clearly distinct composition of PGs from RLBs (Fig. S4A; Fig. 3). Next, we treated WT or RNase L knockout A549 cells with VFS and stained them for G3BP1 and PABP1. Both WT and RNase L knockout A549 cells were able to assemble PGs in response to VFS stimulation (Fig. S4B). In addition, RLBs and PGs are distinct in their timing of assembly, because we observed PGs form rapidly within 10–20 min of VFS exposure, whereas RLBs form over 1–2 h and are preceded by the formation of SGs that then remodel into RLBs (Burke et al., 2020). Overall, these results therefore suggest that RLBs and PGs are distinct SG-like bodies.

Recent studies have proposed a model for SG formation structured around the interplay of a large interaction network of mRNAs and key nucleator proteins enriched in intrinsically disordered regions (IDRs), prion-like domains (PrLDs) and RNA-binding domains (RBDs) (Guillen-Boixet et al., 2020; Sanders et al., 2020; Yang et al., 2020). We used the online tool SLIDER, which predicts whether a protein sequence has long disordered segments (at least 30 consecutive disordered residues), to analyse PG proteins (Peng et al., 2014). We observed an increase in proteins with long IDRs in the G3BP1 IP compared to the IgG pulldown (Fig. 3E). Moreover, using STRING for interaction analysis, we performed *in silico* screening in order to identify which protein domains were most enriched in PGs (Szklarczyk et al., 2019). RBDs and RNA recognition motifs (RRMs) were the most enriched domains within the reported top 10 protein domains identified across three different databases, Pfam, SMART and InterPro (Fig. 3F). Finally, to better characterise the common feature of the 110 PG proteins, we performed a Gene Ontology (GO) enrichment analysis with Cytoscape using ClueGO and the online platform Metascape (Zhou et al., 2019). This confirmed the overrepresentation in the PG composition of RBPs, for example factors involved in RNA splicing (GO:0008380, GO Biological Processes), RNA localization (GO:0006403), mRNA catabolic process (GO:0006402), and in focal adhesion (GO:0005925, GO Cellular Components) and ribonucleoprotein granule (GO:0035770). KEGG and Reactome pathway analysis was carried out to identify possible pathways activated by VFS stimulation (Fig. 3G). Interestingly, metabolism of RNA (R-HSA:8953854) and cell-to-cell communication (R-HSA:1500931) were identified among the predicted pathways, fitting with a paracrine mechanism of induction. We also identified pathways induced by stresses such as senescence and BRAF signalling (R-HSA:6802952) and other paracrine pathways such as FGFRs (R-HSA:5654738) (Table S4).

### PG assembly results in the condensation of functional classes of mRNAs similar to those found in SGs

**Fig. 4.**
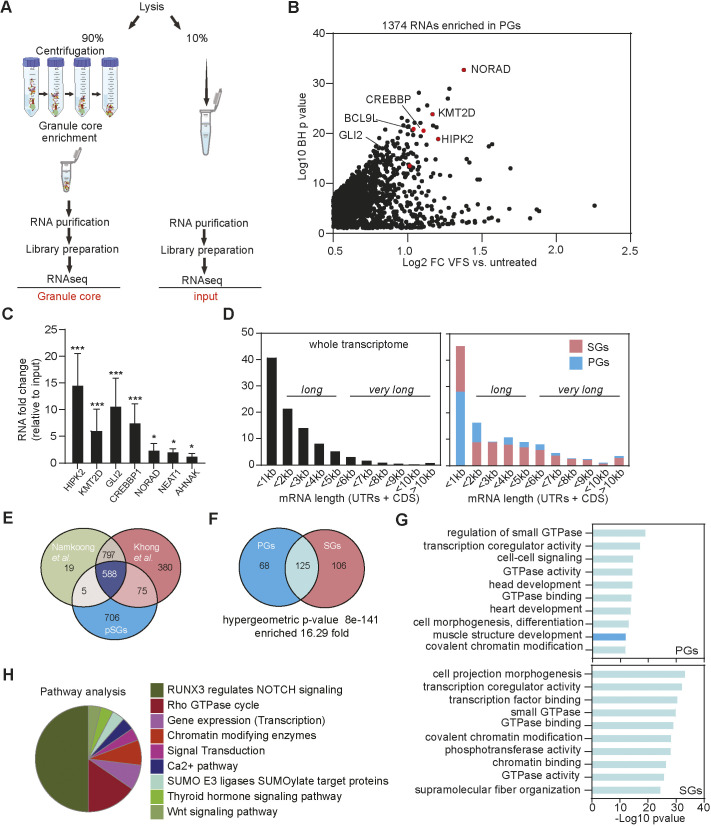
**Transcriptomic analysis reveals differences between VFS-induced PGs and ARS-induced SGs.** (A) Schematic representation of the procedure for total and PG transcriptome analysis in VFS-treated or untreated cells. (B) Volcano plot showing statistically significantly enriched differentially expressed RNAs (log_2_ fold change of VFS versus non-treated) in PGs. RNAs in red correspond to those validated by qPCR in C. BH, Benjamini–Hochberg. (C) Transcript levels of PG resident mRNAs were quantified via RT-qPCR relative to untreated and normalized to the individual total level of each RNA. Error bars represent s.e.m. (*n*=3), and statistical significance is shown above the bars, *P<0.05, ***P<0.005 (one-way ANOVA). (D) Comparison of the mRNA length for transcripts enriched in PGs and arsenite-induced SGs (from Khong et al., 2017) compared to total transcriptome distribution. CDS, coding DNA sequence; UTR, untranslated region. (E) Venn diagram comparison of mRNAs enriched in PGs and arsenite-induced SGs (from Khong et al., 2017; Namkoong et al., 2018). (F) Venn diagram comparison of the GO terms (molecular function and biological process) enriched in the mRNAs enriched in PGs or SGs upon VFS-stimulation or sodium arsenite (ARS) treatment (from Khong et al., 2017). (G) Comparison of the top 10 GO terms enrichment (molecular function and biological process) for mRNAs enriched in PGs (mid blue) or SGs (pink). GO terms overlapping both conditions are in light blue. (H) Clustering by functional pathways identified from GO analysis (KEGG/Reactome) of the mRNAs enriched in VFS-treated versus non-treated U2OS cells.

Previous studies have underpinned the roles of mRNA species in the assembly of SGs, showing that they support the network of weak and transient interactions required during condensation (Van Treeck et al., 2018). In addition, RNAs can condense on their own, resulting in SG-like condensates, with almost identical mRNA contents, in the absence of protein (Van Treeck et al., 2018). Moreover, although SGs are thought to sequester a wide variety of cytoplasmic mRNAs, specific transcripts excluded from sequestration can drive a specific translational programme to adapt to stress (Khong et al., 2017; Namkoong et al., 2018). Thus, to further characterise the PG-SG differences, we analysed the RNA contents of PGs using RNA-seq. To this end, we used the granule-enriched fraction, given that it had previously been shown to accurately reflect the RNA content of isolated SGs (Namkoong et al., 2018). As outlined in Fig. 4A, U2OS GFP-G3BP1 cells were treated with VFS, or untreated, lysed and following preparation of the granule-enriched fraction, RNAs were then purified, sequenced by the Illumina method (PG transcriptome), and compared to the total RNAs (total transcriptome).

Total transcriptome analysis identified 15698 transcripts overall and 7450 were selected for further analysis with a significant Benjamini–Hochberg *P*-value <0.05 (Table S5). We analysed the coverage of the different RNA species present in the RNA-seq dataset and did not observe any rRNA accumulation, confirming that our samples were efficiently rRNA depleted, and, as expected, the majority of the RNA species identified, 95.9%, were protein coding, with long non-coding RNAs (lncRNAs) representing 2.5% and the remaining corresponding to small nucleolar RNAs (snoRNAs), small nuclear RNAs (snRNAs) and microRNAs (miRNAs) (Fig. S5A). We further looked at different features of RNA, such as length and GC content. We observed that 41% of mRNAs were less than 1 kb long and the majority of RNAs (67.3%) contained less than 50% GC content (Fig. S5B). Furthermore, we performed a differential expression analysis comparing the RNAs expressed (transcriptome) in the VFS versus untreated cells used as control; we identified 712 RNAs that were upregulated at least 0.5 log_2_ fold change above the counts per million (CPM) of RNAs in VFS-treated cells relative to the untreated cells, and 2510 RNAs were downregulated (Fig. S5C, Table S6). This analysis revealed that the majority of the RNAs were downregulated in VFS versus the control, including many lncRNAs such as NORAD and NEAT1 (Fig. S5D). Both up- and downregulated miRNAs could be identified, whereas the majority of lncRNAs were downregulated. We validated some of the transcript RNAs via RT-qPCR analysis (Fig. S5E), confirming the inhibition of gene expression induced by VFS treatment, and observed a strong downregulation of BCL9 L, HIPK2, CREBBP1, GLI2, NORAD and NEAT1. Interestingly, the majority of mRNAs that were not downregulated encoded ribosomal proteins previously shown to exhibit a stable and/or long half-life, such as RPS18 and RPL9 (Fig. S5E).

Functional GO analysis (molecular function and biological process) revealed that the majority of upregulated RNAs are involved in the oxidative phosphorylation (GO:0006119), such as ATP synthase, cytochrome *c* oxidase and NADH-ubiquinone oxidoreductase, mitochondrial (GO:0006839) and translation-like ribosome components (KEGG pathway hsa03010) (Fig. S6A). We further compared GO terms enrichment for the top 500 significant RNAs identified in VFS- or arsenite-treated U2OS cells and U2OS G3BP1-GFP cells (Fig. S6B,C, Table S7) (Khong et al., 2018). Analysis of the most enriched GO terms revealed a small overlap, suggesting that the overall pathways activated by these two types of stresses are different. The comparison of the top 10 summary GO terms enrichment showed that the VFS induces an effect mainly at transcription regulation level (GO:0003712, GO:0008134, GO:0001227) in contrast to mRNA metabolism for arsenite (GO:0022613, GO:0006402, GO:0031145).

Next, analysis of the PG transcriptome identified 1374 transcripts with a 0.5 log_2_-fold change above the background (untreated cells) (Fig. 4B; Table S8). As expected, most of the RNAs identified encoded protein (92.3%), and the remaining transcripts were identified to be 3.7% lncRNAs, 1.5% miRNAs and the rest RNAs such as snoRNA, and processed and unprocessed pseudogenes. We confirmed by RT-qPCR some of the most enriched RNAs identified in PGs including HIPK2, CREBBP1, GLI2 and KMT2D, whereas lncRNAs such as NORAD, NEAT1 and AHNAK were only slightly enriched (Fig. 4C).

We next analysed whether enriched transcripts presented common features such as length or GC contents that could indicate their ability to form large RNPs. We observed that only 28.5% of mRNAs in PGs were ‘short’ (less than 1 kb), with the majority of mRNAs enriched, 46.7%, ‘long’ (between 1 and 5 kb), and 24.8% were ‘very long’ (above 5 kb). This was surprisingly different compared with the mRNAs enriched in canonical SGs, which for the majority were ‘short’ 45.7%, only 33.9% were ‘long’ and just 20.4% were ‘very long’ mRNAs (Khong et al., 2018) (Fig. 4D). The majority of RNAs enriched in PGs, 91.2%, displayed less than 50% GC content, reflecting not very structured RNAs.

The comparison of RNAs enriched in arsenite-induced SGs and PGs revealed only 50.7% overlap between the two datasets (Fig. 4E). Therefore, we compared the GO terms enrichment for SG and PG resident RNAs in U2OS GFP-G3BP1 cells (Fig. 4F). This revealed a significant overlap between the two classes of condensates (16.3-fold), suggesting that although the 50% of RNAs enriched in PGs were different from the RNAs enriched in SGs, they shared significant functional roles (Fig. 4F,G; Table S9). The top 10 GO terms enriched in PGs, involving the small GTPase-mediated signal transduction (GO:0007264) and cell junction organization (GO:0034330), were also significantly enriched in the 125 GO terms common between PGs and SGs, and vice versa (Fig. 4G; Table S10). Finally, analysing the most enriched pathways (Reactome and KEGG), using the filter at *P*-value <0.01, highlighted that the cell-cell communication pathway (RUNX3-NOTCH) represented 50% of the enriched GO terms (Fig. 4H; Table S11). Overall, these results suggest that VFS treatment induces global changes in the U2OS transcriptome that are different from those observed during arsenite-induced oxidative stress, but that similar functional classes of mRNAs are enriched in SGs and PGs.

### N6-methyladenosine modified RNAs and ARE motif are enriched in PGs

**Fig. 5.**
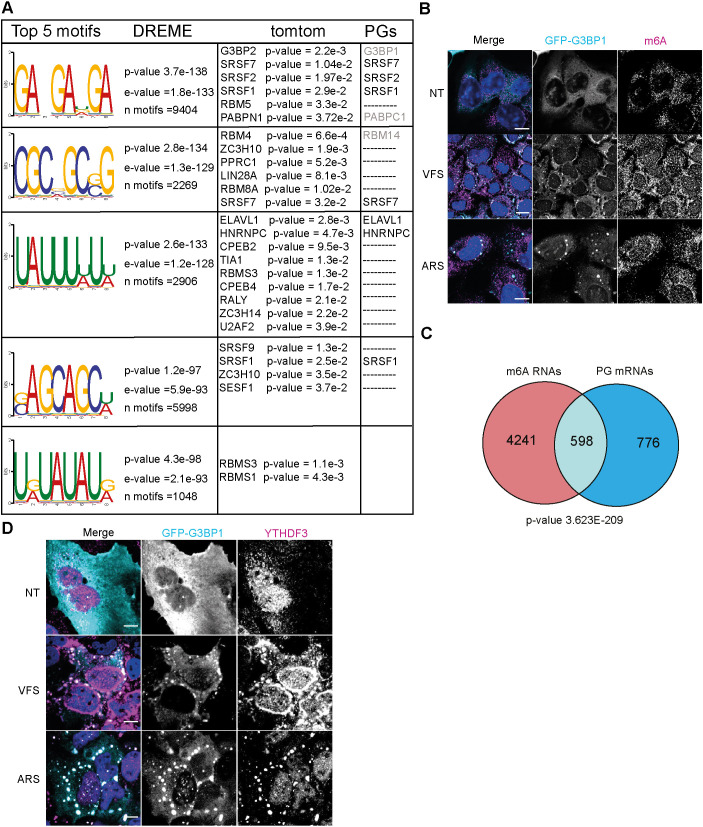
**N6-methyladenosine modified RNAs are enriched in PGs.** (A) Top 5 RNA motifs identified from DREME analysis of the PG transcriptome, with corresponding putative RBP targets identified from Tomtom analysis of these motifs and the corresponding proteins identified by proteomic analysis as PG components. (B) U2OS GFP-G3BP1 cells were stimulated for 1 h with VFS or 0.5 mM sodium arsenite (ARS) prior to fixation and the formation of PGs or SGs analysed by confocal microscopy. Non-treated (NT) cells were used as controls. Cells were immunostained against GFP (cyan) and m6A (magenta). Nuclei were stained with DAPI (blue). Scale bars: 10 μm. (C) Venn diagram comparison of 1374 RNAs identified in PGs and 4839 m6A-edited mRNAs localised to SGs (from Anders et al., 2018). The representation factor shows the enrichment over the expected, and the *P*-value (two-tailed unpaired *t*-test) is based on the cumulative distribution function of the hypergeometric distribution of the dataset over the human genome. (D) The presence of G3BP1 granules was assessed as in B. Cells were stained with GFP (cyan) and YTHDF3 (magenta). Nuclei were stained with DAPI (blue). Scale bars: 10 μm. Images in B and D are representative of three experiments.

To investigate whether specific RNA motifs were enriched in the PG transcriptome, we selected the top 500 most significantly enriched mRNAs and analysed motif enrichment using MEME and DREME (Bailey et al., 2009). The top five motifs enriched in the PGs are displayed in Fig. 5A, with the corresponding ‘E-value’ for each identified motif, and included GA-GA[U/G/A]GA, CGC[G/C/A]GC[G/C]G, UAUUU[U/A]U[U/A], [G/C]AGCAGC[U/A] and U[G/A]UAUAU[G/A]. We then correlated these with RBP binding using the MEME built-in Tomtom tool to identify putative RBPs previously described to bind each specific given motif (Fig. 5A). Interestingly, this revealed that some of the RBPs identified as resident PG proteins by our proteomic analysis are targets of the enriched RNA motifs, such as ELAVL1, SRSF2 and SRSF7, and G3BP2 which shares a similar binding motif to G3BP1 (Edupuganti et al., 2017).

Additionally, it has been proposed that N6-methyladenosine (m6A) RNA modifications play a role in triaging mRNAs from the translatable pool into SGs (Anders et al., 2018). Upon binding of these target mRNAs, their m6A reader proteins, the YTH domain family proteins (YTHDFs), undergo LLPS and promote SG formation (Elbaum-Garfinkle, 2019; Yang et al., 2020). This overlap points to a possible link between m6A modification, translation under stress conditions and possible recruitment to SGs (Zhou et al., 2015). To determine whether m6A-modified RNAs were enriched in PG, we performed immunofluorescence in U2OS GFP-G3BP1 cells treated either with VFS or arsenite and analysed the cellular distribution of m6A mRNAs using a specific antibody previously used to pull down and identify these mRNAs (Anders et al., 2018). As shown in Fig. 5B, both arsenite and VFS treatment resulted in colocalization of m6A signal with G3BP1. Next, we compared the list of m6A-modified mRNAs localised to SGs following arsenite treatment from Anders et al. (2018) with PG resident mRNAs (Fig. 5C). We observed a 3.8-fold enrichment between the two groups, with a significant overlap (hypergeometric *P*-value=3.6×10^–209^), confirming that a significant proportion of PG mRNAs could be m6A-modified. As m6A-mRNA triage is thought to be driven by YTHDF3, which localises to SGs during stress, we performed immunofluorescence to investigate its cellular localisation following VFS treatment. This confirmed that YTHDF3 colocalised with G3BP1 in the PGs (Fig. 5D). We also notice that YTHDF3 displayed a peculiar distribution around the nucleus not observed with arsenite-induced stress. Overall, these data suggest that like SGs, PGs might condensate m6A-modified mRNAs and their reader protein YTHDF3.

### PGs assembly is coupled to translational shut-off and activation of stress signalling pathways

**Fig. 6.**
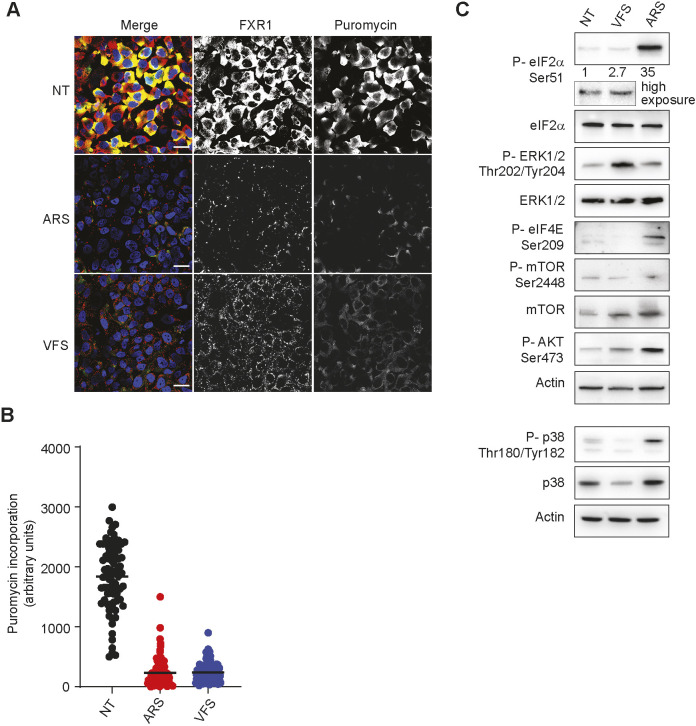
**PG assembly is associated with global shut-off in translation and activation of intracellular signalling pathways.** (A) U2OS cells stimulated for 1 h with VFS or 0.5 mM sodium arsenite (ARS) were incubated with 10 μg/ml puromycin to label nascent polypeptide chains prior to fixation. Non-treated (NT) cells were used as a control. Puromycin-labelled chains were visualised by immunostaining against puromycin (green), and PG or SG cells were detected by immunostaining against FXR1 (red). Nuclei were stained with DAPI. Scale bars: 100 μm. (B) Representative scatter plots of *de novo* protein synthesis measured by fluorescence intensity of the puromycin signal (*n*=3). Lines indicate the mean values. (C) Representative western blot analysis (*n*=3) of cells stimulated as in A. Antibodies used are indicated to the left. The levels of phosphorylated eIF2α are shown, normalised to levels in non-treated cells.

Canonical SGs are assembled in response to translational stalling that can be triggered by eIF2α-dependent or -independent pathways and result in the storage of the bulk of cytoplasmic mRNAs until stress is resolved. Thus, we investigated whether PG assembly is also coupled to translational inhibition. To this end, we determined global translational efficiency using single cell analysis by measuring the incorporation of puromycin, a tRNA structural mimic that specifically labels actively translating nascent polypeptides and causes their release from ribosomes. Puromycylated native peptide chains are then detected with anti-puromycin antibodies and confocal microscopy (Fig. 6A). As expected, quantification of the puromycin signal intensity revealed a strong decrease in protein synthesis following treatment with arsenite in U2OS cells (Fig. 6A,B). Treatment of U2OS cells with VFS also resulted in impaired puromycin incorporation and inhibition of protein synthesis (Fig. 6B). Therefore, assembly of PGs, like SGs, is coupled to translational shut-off. To understand the cellular signalling pathways responsible for communicating this stress, we analysed the activation of several stress-associated signalling proteins using immunoblotting (Fig. 6C). As expected, arsenite treatment resulted in the phosphorylation of eIF2α, which occurs through the kinase HRI. VFS treatment also induced eIF2α phosphorylation, relative to the total level of total eIF2α protein level (Fig. 6C). None of the hallmarks of eIF2α-independent signalling activation via mTOR could be detected, with no increase in phosphorylation of AKT and mTOR. In contrast, analysis of the MAPK signalling pathway revealed that VFS treatment resulted in a strong increase in ERK phosphorylation, but not eIF4E or p38, although we noted a decrease in both total and phosphorylated p38 levels. These results suggest that PG assembly is coupled to translational inhibition and activation of stress-related signalling pathways such as eIF2α and ERK1/2. To understand whether these pathways act upstream or downstream of PG assembly, we treated mouse embryonic fibroblast (MEF) cells expressing WT or a non-phosphorylatable mutant of eIF2α (MEF S51A; Jiang et al., 2003) with VFS. Whereas arsenite-induced SG assembly was abolished in S51A MEFs as expected, both WT and S51A MEFs assembled PGs in response to VFS treatment (Fig. S7A). Quantification of the number of cells displaying PGs revealed that impairing eIF2α phosphorylation only reduced PG assembly to 58% (Fig. S7B). This suggests that although it contributes to PG formation, eIF2α phosphorylation is not essential for their assembly, unlike for SGs. Next, U2OS cells were treated with the ERK1/2 inhibitor SCH772984 (Royall et al., 2015). Pharmacological inhibition of ERK1/2 did not impair the VFS-induced assembly of PGs (Fig. S7C). Therefore, these results suggest that ERK activation may result from the VFS stimulation and PG formation, rather than drive PG assembly itself.

### PGs play a role in antiviral response

**Fig. 7.**
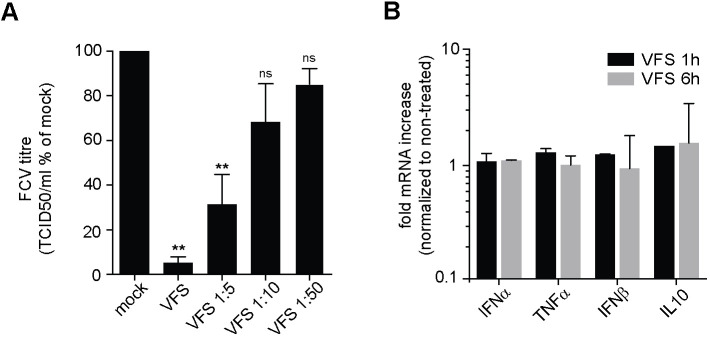
**VFS treatment impairs FCV replication in CRFK cells.** (A) CRFK cells were stimulated for 1 h with decreasing amounts of VFS and infected with FCV at an MOI of 1. The cells were incubated for 12 h, and the viral titre was estimated by a TCID_50_ assay. Error bars represent s.d. Three separate experiments were analysed by standard two-tailed paired *t*-test (**P<0.01; ns, not significant). (B) Transcript levels of indicated mRNAs were quantified via RT-qPCR in CRFK cells following stimulation with VFS for 1 or 6 h and normalized to non-treated cells. Error bars represent s.e.m. (*n*=3).

The formation of PGs was first observed during infection of Crandell-Rees feline kidney (CRFK) cells with FCV in bystander cells. Recent work has proposed that SGs exert antiviral activities by providing a platform for antiviral signalling through condensing IFN signalling molecules (Eiermann et al., 2020). Therefore, we hypothesised that PG assembly could be part of paracrine signalling to reduce or prevent the spread of FCV to uninfected bystander cells. To test this hypothesis, CRFK cells were treated with increasing amounts of VFS, and then infected with FCV for 6 h at MOI 2, and viral titre measured using TCID_50_ assays. FCV replication was impaired by treatment of CRFK with undiluted VFS, with 95% reduction in infectious titres, whereas dilution of VFS failed to prevent viral replication (Fig. 7A). Finally, we tested whether this impairment of viral replication could be explained by the triggering of antiviral factors. Following 1 or 6 h of treatment with VFS, total RNA was extracted from CRFK cells and the levels of IFN-α/β, TNFα and IL10 measured by qPCR. Compared to untreated control, VFS did not induce IFN-α/β, TNFα or IL10 expression (Fig. 7B). These results suggest that the VFS-induced reduction in viral replication may be caused by translational shut-off rather than activation of antiviral signalling in cells assembling PGs.

## DISCUSSION

We have identified novel SG-like granules induced in bystander cells during FCV infection. Our results support a model in which paracrine signalling during infection results in the activation of stress-related intracellular signalling, including ERK and eIF2α pathways, and a shut-off of translation. This is coupled to the assembly of SG-like condensates we named PGs. PGs share some characteristics with SGs, PBs, RLBs and PSPs, but exhibit their own specific nature. When compared to canonical SGs, PGs display differences in granule size, number per cell and dynamics. Resident PG proteins are, like for SGs, enriched in RNA-binding proteins and proteins with disordered domains, yet PGs condense distinct functional classes of proteins, and lack many of the eIFs found in SGs. Moreover, in contrast to SGs, RNAs found in PGs are significantly longer and their identity is different, yet similar functional classes of mRNAs are enriched within SG and PG. PG-resident RNAs are enriched in RNA motifs recognised by RNA-binding proteins we identified in PGs, such as ELAVL1, SRSF2 and SRSF7, and G3BP2. This raises the possibility that these proteins could play a role during the PG condensation process by driving RNA-protein interactions.

The analysis of the PG assembly-disassembly dynamic revealed that although their assembly followed a similar pattern to that of arsenite-induced SGs, they disassembled more rapidly, with complete clearance within 10 min (Fig. S1). This behaviour is reminiscent of cold-shock SGs (Hofmann et al., 2012). In mammalian cells, following exposure to hypothermia, the PERK (EIF2AK3)-mediated phosphorylation of eIF2α and inhibition of mTOR signalling impairs the assembly of preinitiation translation complexes. AMP-activated protein kinase (AMPK) signalling is further activated to orchestrate cellular adaptation to these stressful conditions, resulting in protein synthesis slow-down and triggering SG assembly and cell survival (Hofmann et al., 2012; Roobol et al., 2009). Although cold shock-induced SGs form over the course of several hours, they disassemble within minutes when cells are returned to ideal growth temperatures. Cold shock-induced SGs condense poly(A) mRNAs and known SG resident proteins such as eIF3B, eIF4G, eIF2α, G3BP1, G3BP2, PABP1, HuR and TIA1, confirming that they are bona fide SGs; however, they are smaller in size and more abundant than arsenite-induced SGs. Our study did not reveal activation of AMPK signalling targets, and PGs lack most eIF components, suggesting that PGs and cold shock-induced SGs are associated with distinct pathways of cellular adaptation to stress (Fig. 6C).

In addition to PKR activation, the 2′,5′-oligoadenylate synthetase (OAS)-RNase L pathway can also limit host cell translation in response to dsRNA stimulation or viral infection. Whereas activation of PKR promotes SG assembly, RNase L signalling inhibits assembly of chemically induced SGs and actively promotes SG disassembly (Burke et al., 2019, 2020). This results in a translational shut-off that is independent of PKR and phospho-eIF2α, and the assembly of RLBs (Burke et al., 2020). Importantly, although RLBs are sensitive to CHX treatment, they do not require G3BP1 for their assembly, and thus are distinct from SGs, sharing this property with PGs (Fig. S6). However, these results have been challenged by another study suggesting that the specific RNase L ligand 2-5A, and the associated RNase L stimulation, results in the assembly of antiviral SGs (avSGs) rather than RLBs (Manivannan et al., 2020). In brief, avSGs are different from RLBs as they require G3BP1 and activate PKR (Manivannan et al., 2020). These avSGs contain several components of the innate immune pathways, including RIG-I, PKR, OAS and RNase L, and G3BP1 interacts with RIG-I and PKR. Moreover, their assembly is required for IRF3-mediated production, and the absence of avSGs sensitized the cells to infection. Therefore, avSGs have been proposed to provide a platform for efficient interaction of RNA ligands and pattern recognition receptors (PRRs) to help mount the antiviral state. Comparing the list of PG resident proteins to the RLB proteome revealed that although PG and RLB share a lack of requirement for G3BP1, they contain different proteins and are thus likely to fulfil distinct cellular functions (Fig. S6) (Burke et al., 2020). In addition, PGs are insensitive to RNase L depletion, which is essential for RLB assembly.

During infection by viruses, several viral products can act as pathogen-associated molecular patterns recognized by PPRs to amplify the IFN response and create an antiviral state (Kawai and Akira, 2006). Among these, PKR is a major player in antiviral innate immunity. PKR is activated upon binding to viral double-stranded RNA (dsRNA), triggering the integrated stress response (ISR), global translation inhibition and SG formation (Balachandran et al., 2000). In addition, PKR also acts through innate immune signalling pathways such as nuclear factor-κB (NF-κB), leading to the production of pro-inflammatory cytokines. Therefore, viruses have evolved various strategies to suppress PKR activation and thereby block the ISR and SG formation. These strategies include PKR degradation, PKR inhibition by viral proteins or RNAs, and shielding of dsRNA by viral proteins (Eiermann et al., 2020; Jan et al., 2016). Moreover, many viruses counteract SG formation directly, suggesting that SGs themselves, and not only the ISR, are the target. These strategies include encoding proteases that cleave G3BP1, sequestration of SG resident proteins by viral proteins or viral RNAs, or repurposing of SG proteins for viral replication (Gaete-Argel et al., 2019; McCormick and Khaperskyy, 2017; Poblete-Duran et al., 2016). A large body of evidence, including functional analysis of virus deletion mutants that abrogate SG formation, or SG composition, suggests that SGs themselves contribute to antiviral response. The ability of G3BP1 to inhibit enterovirus replication is directly linked to SG formation, and studies proposed that this is mediated by PKR recruitment to SGs by G3BP1, which potentiates PKR and impairs viral replication (Reineke and Lloyd, 2015). Additional support to an antiviral role for SGs is provided by reduced PKR activation following pharmacological or genetic disruption of SG formation (Burgess and Mohr, 2018; Manivannan et al., 2020; Ng et al., 2013; Oh et al., 2016; Onomoto et al., 2012; Reineke et al., 2015; Reineke and Lloyd, 2015).

Several other antiviral proteins, including RIG-I, MDA5, OAS, RNase L, Trim5, ADAR1, ZAP, cGAS and RNA helicases, can be recruited to SGs during infection, suggesting that these are antiviral SGs (avSGs) with a role in antiviral signalling (Onomoto et al., 2012; Thulasi Raman et al., 2016; Yoo et al., 2014). avSGs are essential for IFN signalling in response to several (–) ssRNA viruses, as their loss or inhibition results in increased replication and impaired antiviral response. The colocalization of viral RNAs and PRRs further suggested that these provide a platform for non-self sensing and prime IFN production (Ng et al., 2013; Oh et al., 2016; Yoshida et al., 2015). Moreover, the antiviral activity of the zinc-finger antiviral protein (ZAP) correlates with its recruitment to SGs, inhibiting the replication of several viruses through exosome-mediated viral RNA degradation (Law et al., 2019; Leung et al., 2011). Overall, this implicates SGs in antiviral signalling. Our study links the formation of PGs to antiviral activity, given that PG assembly is associated with impaired FCV replication. However, proteomic analysis of PG composition did not reveal the accumulation of specific PPRs or RLRs. PG resident proteins clustered into functional categories related to stresses such as senescence and BRAF signalling, paracrine pathways such as FGFR, DNA damage and rRNA processing. In addition, the analysis of the transcriptional reprogramming associated with PG assembly did not highlight typical IFN responses, and most differentially expressed genes were functionally clustered in categories related to oxidative phosphorylation, and mitochondrial and translation-like ribosome components. Therefore, we speculate that PGs may restrict viral replication through mechanisms distinct from the non-self-sensing platform hypothesis proposed above for SGs and avSGs.

To date, there are only limited examples of paracrine inducers of SG assembly. Angiogenin, an angiogenic factor released by tumour cells, acts as a secreted stress-activated ribonuclease, producing tRNA-derived stress-induced RNAs (tiRNAs) (Emara et al., 2010). tiRNAs are then recognized as PKR ligands, promoting translational repression and inducing SG assembly (Emara et al., 2010). In addition, prostaglandins are secreted neuroinflammatory molecules, produced after environmental insults to the brain and associated with neurodegeneration (Figueiredo-Pereira et al., 2014). 15-deoxy-Δ12,14-prostaglandin J2 (15-d-PGJ2) is the most reactive prostaglandin; it covalently modifies eIF4A, and causes eIF2α phosphorylation to block translation initiation (Campo et al., 2002; Kim et al., 2007; Marcone and Fitzgerald, 2013). As a result, 15-d-PGJ2 is an endogenously produced trigger of SG assembly and activates the ISR in a cell-nonautonomous manner (Tauber and Parker, 2019). In preliminary experiments, we could not detect the production of angiogenin or 15-d-PGJ2 following VFS stimulation (data not shown). Therefore, we do not rule out several possible origins for the paracrine messenger: nucleic acid (RNA fragment of viral origin, cellular miRNAs), proteins (cytokines or signalling proteins) or more complex structures such as exosome particles. Assessing these possibilities, and whether infection with other viruses induces the formation of PGs, should be the focus of further studies. Overall, our work expands our understanding of the role of biocondensates in eukaryotes, describing a novel type of cytoplasmic RNP granule formed in response to viral infection and associated with control of viral replication.

## MATERIALS AND METHODS

### Cells and viruses

CRFK cells [European Collection of Cell Cultures (ECACC)] were grown in minimum essential medium (MEM, Gibco #31095-029) containing L-glutamine (Gibco) and 1% nonessential amino acids (Gibco), and supplemented with 10% fetal bovine serum (FBS, Gibco) and 1% penicillin-streptomycin, in a 5% CO_2_ environment. U2OS and A549 cells were grown in DMEM (Dulbecco's Modified Eagle's Medium, Gibco #41966), supplemented with 10% (v/v) fetal bovine serum (FBS, Gibco), glutamine and 1% penicillin-streptomycin (Gibco) in a 5% CO_2_ chamber. GFP-G3BP1 expressing, G3BP1/2 double knockout U2OS, UBAP2L knockout HeLa, MEFs S51A and RNase L knockout A549 cells have been described elsewhere (Brocard et al., 2020; Burke et al., 2020; Cirillo et al., 2020). Arsenate, SCH772984, puromycin and cycloheximide were all purchased from Sigma. All inhibitors were added to the cell culture media and incubated at 37°C for the indicated times. For viral infections, the FCV strain Urbana was used as previously described at a multiplicity of infection (MOI) equal to 1, and at 0.2 MOI for VFS generation (Humoud et al., 2016). After 1 h the inoculation was removed, and the cells were incubated with fresh medium for 5 h post infection (h.p.i.).

### Virus-free supernatant preparation

CRFK cells (2×10^6^) were seeded in 10 cm dishes and incubated overnight. On the following day, the cells were infected with FCV at 0.2 MOI for 1 h and the supernatant was collected 5 h.p.i. and spun at 500 ***g*** at 4°C (Micro 22R, Hettich) for 30 min. After centrifugation, supernatant was filtered through a 0.2 μm Millex filter. The FCV particles were removed via precipitation by adding 0.2 M solid NaCl and 10% PEG3350 and incubated overnight at 4°C in constant rotation. Next, the samples were first centrifuged for 60 min at 500 ***g*** (Micro 22R, Hettich) at 4°C and then at 13,400 ***g*** for 4 h 30 at 4°C using an SW41Ti rotor (Beckman). The supernatant was then UV inactivated at a wavelength of 254 nm for 4 min (three times) using a crosslinker (Stratalinker^®^ UV Crosslinker), filtered through a 0.2 μm Millex filter and stored in −20°C until use.

### Paracrine granule isolation

For PG isolation, we followed the protocol previously described in Brocard et al. (2020). Briefly, G3BP1-GFP-U20S cells were grown overnight in 15 cm dishes to 80–90% confluence and incubated with 8 ml of VFS for 1 h, then washed twice with warm DMEM, scraped out and spun down at 1500 ***g*** for 10 min. The cell pellet was either snap frozen or resuspended in 1 ml of lysis buffer (50 mM Tris-HCl, pH 7.4, 100 mM potassium acetate, 2 mM magnesium acetate, 0.5 mM DTT, 50 μg/ml heparin, 0.5% NP40, 1 complete mini EDTA-free protease inhibitor tablet/50 ml of lysis buffer), lysed using a syringe and a 25G 5/8 needle on ice seven times, and spun at 300 ***g*** for 5 min at 4°C. The supernatant was transferred and centrifuged again at 18,000 ***g*** for 20 min at 4°C. The supernatant was discarded and the pellet was resuspended in 1 ml of SG lysis buffer before spinning again at 18,000 ***g*** for 20 min at 4°C. The pellet was resuspended in 300 μl of lysis buffer to yield the granule-enriched fraction. The IP was performed using a preclearing with 60 μl of Protein A Dynabeads of the granule-enriched fraction for 20–30 min in rotation at 4°C. The supernatant was then incubated with 0.5 μg of anti-GFP antibody by rotating at 4°C overnight. A 500 μl volume of SG lysis buffer was added to each sample before spinning down at 18,000 ***g*** for 20 min at 4°C. The resulting pellet was resuspended in 500 μl of SG buffer and 33 μl of Dynabeads, then incubated for 3 h at 4°C in rotation. The beads were washed once with 1 ml of wash buffer 1 (SG lysis buffer+2 M urea) at 4°C for 2 min, then washed for 5 min with 1 ml of buffer 2 (SG lysis buffer+300 mM potassium acetate) at 4°C, 5 min in SG lysis buffer at 4°C and then seven times with 1 ml of TE buffer (10 mM Tris, 1 mM EDTA). The resuspended beads were sent in TE (10 mM Tris-HCl, pH 8.0, 1 mM EDTA) buffer for MS/MS analysis to the University of Bristol Proteomics Facility.

### Sample preparation for LC-MS/MS analysis

Samples from IP were resuspended in 0.1 M ammonium bicarbonate (ABC) and 0.1% sodium deoxycholate, then reduced and alkylated using 5 mM TCEP and 20 mM chloroacetamide at 70°C for 15 min in darkness. Samples were then trypsinized using 0.25 µg of sequencing-grade modified trypsin (Promega) at 42°C for 4 h. Sodium deoxycholate was removed by phase transfer to ethyl acetate. The resulting tryptic peptides were desalted using in-house StageTips with a 3M Empore SDB-RPS membrane, and dried using vacuum centrifugation. The peptides were reconstituted in 15 µl of Buffer A (0.1% formic acid in water), of which 5 µl was subjected to LC-MS/MS analysis.

### LC-MS/MS analysis

The tryptic peptides were resolved using a Waters nanoACQUITY UPLC system in a single pump trap mode. The peptides were loaded onto a nanoACQUITY 2G-V/MTrap 5 µm Symmetry C18 column (180 µm×20 mm) with 99.5% Buffer A and 0.5% Buffer B (0.1% formic acid in acetonitrile) at 15 µl/min for 3 min. The trapped peptides were eluted and resolved on a BEH C18 column (130 Å, 1.7 µm×75 µm×250 mm) using gradients of 3 to 5% B (0–3 min), 8 to 28% B (3–145 min), and 28 to 40% B (145–150 min) at 0.3 µl/min. MS/MS was performed on a LTQ Orbitrap Velos mass spectrometer, scanning precursor ions between 400 and 1800 m/z (1×106 ions, 60,000 resolution) and selecting the 10 most intense ions for MS/MS with 180 s dynamic exclusion, 10 ppm exclusion width, repeat count=1, and 30 s repeat duration. Ions with unassigned charge state and MH+1 were excluded from the MS/MS. Maximal ion injection times were 10 ms for Fourier Transform (one microscan) and 100 ms for Linear Ion Trap (LTQ), and the automated gain control was 1×10^4^. The normalized collision energy was 35% with activation Q 0.25 for 10 ms.

### Mass spectrometry data analysis

MaxQuant/Andromeda (version 1.5.2.8) was used to process raw files from LTQ Orbitrap, and search the peak lists against the UniProt human proteome database (total 71,803 entries, downloaded 1 December 2018). The search allowed trypsin specificity with a maximum of two missed cleavages, and set carbamidomethyl modification on cysteine as a fixed modification and protein N-terminal acetylation and oxidation on methionine as variable modifications. MaxQuant used 4.5 ppm main search tolerance for precursor ions, and 0.5 Da MS/MS match tolerance, searching the top eight peaks per 100 Da. False discovery rates for both protein and peptide were 0.01 with a minimum seven amino acid peptide length. A label-free quantification (LFQ) was enabled with a minimum of two LFQ ratio counts and a fast LFQ option.

All raw data were analysed with MaxQuant software. Two or more unique peptides were used for protein identification and a ratio count of two or more for label-free protein quantification in all samples. The LFQ intensities were normalised such that at each condition and time point the LFQ intensity values added up to exactly 1,000,000, therefore each protein group value can be regarded as a normalized microshare (performed separately for each sample for all proteins that were present in that sample). The mass spectrometry proteomics data have been deposited to the ProteomeXchange Consortium via the PRIDE partner repository with the dataset identifier PXD021881.

### RNA sequencing and data analysis

The granule-enriched fraction from VFS or NT cells was generated as described above. The granule-enriched fraction pellets were resuspended in 300 µl of lysis buffer. RNA fractions were extracted with TRIzol following the manufacturer's instructions (Invitrogen). The precipitated RNAs were used to generate a cDNA library by Novogene using oligo(dT) beads, and then fragmented randomly in fragmentation buffer, followed by cDNA synthesis using random hexamers and reverse transcriptase. After first-strand synthesis, a custom second-strand synthesis buffer (Illumina) was added with dNTPs, RNase H and *Escherichia coli* polymerase I to generate the second strand by nick translation. The final cDNA library was completed after a round of purification, terminal repair, A-tailing, ligation of sequencing adapters, size selection and PCR enrichment. The library concentrations were quantified using a Qubit 2.0 fluorometer (Life Technologies), and then diluted to 1 ng/μl before checking insert size on an Agilent 2100 instrument and quantified by quantitative PCR (Q-PCR) (library activity >2 nM). Libraries were fed into Illumina machines according to activity and expected data volume.

### Preprocessing

Quality checks were performed via FastQC (version 0.11.4) (http://www.bioinformatics.babraham.ac.uk/projects/fastqc). The Trimmomatic tool (version 0.32) (Bolger et al., 2014) was used for quality trimming and clipping of adapters and repeated sequences. Reads were mapped to the human genome annotation (GENCODE Human GRCh38.p12 assembly genome and comprehensive gene annotation, http://www.gencodegenes.org) using the RNA-seq aligner STAR (version 2.5.5b) (Dobin et al., 2013). The function featureCounts from the R package Rsubread (version 1.30.9) (Liao et al., 2014) was used to assign mapped sequencing reads to genomic features. Genomic features were defined by the tool's in-built NCBI RefSeq annotations for the hg38 genome. The R package org.Hs.eg.db (version 3.6.0) [Marc Carlson (2016) org.Mm.eg.db: Genome wide annotation for Mouse. R package version 3.6.0.], Ensembl (accessed March 2019 via the R package biomaRt) and GenBank (accessed March 2019 via the R package Annotate) were used to annotate the genomic features.

### Differential expression analyses

Differential expression was performed using the R Bioconductor package EdgeR (version 3.22.5) (Robinson et al., 2010). Filtering of lowly expressed genes was performed, independently for each pairwise comparison, by keeping genes with at least 0.25 counts per million (CPM) in at least 50% of all samples involved in the comparison. EdgeR's default normalization was applied. CPM values were fitted to a negative binomial generalised log-linear model (GLM) using empirical Bayes tagwise dispersions to estimate the dispersion parameter for each gene. Differential expression was identified using GLM likelihood ratio tests. A paired design was used when comparing SGs versus Total in treatment and when comparing SGs versus Total in Control, and a two-group design was used when comparing SGs Treatment versus Control and when comparing Total Treatment versus Control.

### Immunofluorescence microscopy

Cells in a 24-well plate were washed with prewarmed Dulbecco's PBS and immediately incubated in 500 µl of fixing solution (4% PFA in PBS) for 15 min at room temperature, and further permeabilised with 500 µl of 0.1% Triton X-100 in PBS for 10 min at room temperature. For the m6A hybridisation only, the fixation was performed by adding 200 µl per well of ice-cold methanol; the plate was then incubated at 20°C for 10 min, and then washed with PBS. Blocking was carried out with 1 ml of blocking solution (1% BSA in PBS) for 1 h at room temperature. Fixed cells were then incubated with primary antibody in 0.5% BSA-PBS for 2 h at room temperature, and washed three times with PBS before the addition of a secondary antibody for 1 h at room temperature in the dark. All antibodies used are listed in Table S12. Finally, cells were washed three times with PBS and mounted on the slide with ProLong Diamond Antifade with 4′,6-diamidino-2-phenylindole (DAPI; Life Technologies, #P36966). Confocal images were acquired on a Nikon Ti-Eclipse A1M microscope fitted with a 60× oil immersion objective using 488 nm, 561 nm and 405 nm laser excitation lines.

### Preparation of RNA samples and RT-qPCR

Total RNA was extracted using TRIzol (Invitrogen) following the manufacturer's instructions and then subjected to reverse transcription (Primer Design nanoScript v2 kit) with oligo(dT)15 and random primers following the manufacturer's protocol. Subsequently, real time PCR was performed using specific primers listed in Table S13. The samples were analysed in triplicate with SYBR GREEN dye (Primer Design Precision Master mix) on an ABI StepOnePlus quantitative PCR instrument (Applied Biosystems). The comparative Ct method was employed to measure amplification of specific mRNAs versus the total level of β-actin or tubulin where indicated.

### Western blotting

To prepare total cell extracts, GFP U2OS cells were washed twice with cold PBS and then lysed in high salt buffer [50 mM Tris-HCl, pH 7.5, 350 mM NaCl, 1 mM MgCl_2_, 0.5 mM EDTA, 0.1 mM EGTA and 1% (v/v) Triton X-100]; protease inhibitor cocktail was added to the lysis buffer just before use. Lysates were incubated on ice for 30 min, then centrifuged at 4°C, at 10,000 ***g***, for 10 min and the supernatant collected. Protein concentrations were determined using Bradford reagent (Bio-Rad). The lysates were heated at 95°C for 5 min in sample buffer [62.5 mM Tris-HCl, 7% (w/v) SDS, 20% (w/v) sucrose and 0.01% (w/v) Bromophenol Blue] and subjected to polyacrylamide gel electrophoresis and electrophoretic transfer to nitrocellulose (for eIF2alpha blot) or PVDF membranes. Membranes were then blocked in Tris-buffered saline (TBS)-Tween 20 containing 5% (w/v) skimmed milk powder for 30 min at room temperature. The membranes were probed with the primary antibody indicated below in 3% BSA overnight at 4°C, followed by incubation with the appropriate peroxidase-labelled secondary antibodies (Dako) and chemiluminescence development using the Clarity Western ECL Substrate (Bio-Rad). All antibodies used are listed in Table S12. The results were acquired using the VILBER imaging system.

### FRAP and time lapse

GFP-G3BP1 U2OS cells were seeded on glass coverslips in 24-well plates the day before the experiment. The following day, the cells were treated with either VFS or arsenite for 1 h at 37°C before being subjected to FRAP. The cells were imaged using a Nikon AR1 confocal microscope and maintained at 37°C and 5% CO_2_ during imaging acquisition. FRAP experiments were performed as follows: the region of interest (ROI) was defined as the approximate size of a granule (∼2 µm) for bleaching and acquisition area (BL), and a similar ROI size was then determined outside the cells, to account for background (BG). A third ROI was defined around a granule used as a reference for the intensity (REF) to correct the bleaching during image acquisition. The bleaching was carried out with full laser power for 30 s and the acquisition of each ROI was set at every 3 s. The background noise was subtracted from each given time point [BL_corr1(t)=BL(t) – BG(t) and from the REF_corr1(t)=REF(t) – BG(t)], then in order to calculate the normalized corrected bleach value the following formula was applied: BL_corr2(t)=BL_corr1(t)/REF_corr1(t)=[BL(t) – BG(t)]/[REF(t)-BG(t)], before final normalisation to the mean of the prebleach intensity BL_corr3(t)=BL_corr2(t)/BL_corr2(prebleach). The curve for the exponential GFP recovery was determined using GraphPad Prism 8.

### Ribopuromycylation assay

Quantification of *de novo* protein synthesis was performed as described in Brocard et al. (2020). U2OS cells were treated with 10 μg/ml puromycin (Sigma) for 5 min at 37°C, then with 180 μM emetine (Sigma) for 2 min at 37°C. Coverslips were then washed three times with prewarmed DMEM and fixed with 4% PFA in PBS for 10 min at room temperature. Fluorescence intensities were quantified by using the Image J software package Fiji.

### Statistical analysis

Statistical analyses were performed using the GraphPad Prism software, with all experiments being performed with a minimum of three biological replicates, unless indicated otherwise in the figure legends. Statistical significances were calculated by performing one- or two-way ANOVAs or as indicated in the figure legends (****P<0.0001, ***P<0.001, **P<0.01; n.s., not significant).

## Supplementary Material

Supplementary information

Reviewer comments
