# Supplementary material for: Novel stress granule-like structures are induced via a paracrine mechanism during viral infection
Source: J Cell Sci. 2022 Mar 2;135(4):jcs259194. doi: 10.1242/jcs.259194 (PMC8976915; doi:10.1242/jcs.259194)
Supplement: Supplementary information [file joces-135-259194-s1.pdf]

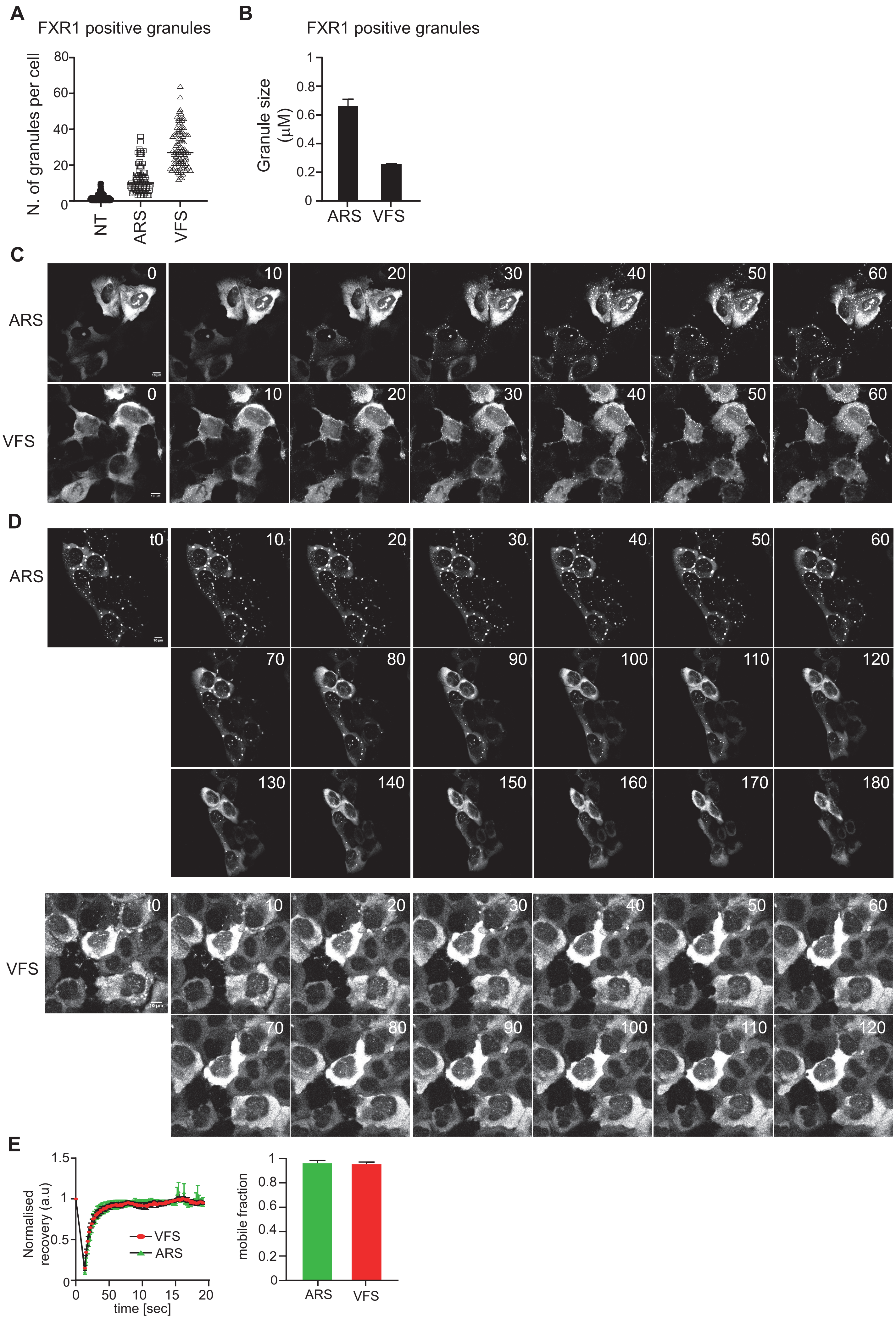

Journal of Cell Science • Supplementary information

Fig. S1. PG and SG have different kinetic of SG assembly and disassembly. (A) and (B) U2OS GFP-G3BP1 cells were stimulated for 1h with VFS or 0.5mM sodium arsenite (ARS) prior to fixation and the formation of stress granules analysed by confocal microscopy using G3BP1 or FXR1 as PG/SG markers. Non-treated (NT) cells were used as controls. Nuclei were stained with DAPI (blue). Representative bar plot (n=3) of the number of FXR1 granules per cells displaying FXR1 foci **(A)** and the average granule size **(B)**, mean ± SD for 100 FXR1-positive cells analysed across at least 10 acquisitions. **(C)** and **(D)** Analysis of SG assembly and disassembly kinetics using time-lapse confocal analysis. **(C)** U2OS GFP-G3BP1 cells were stimulated for 1h with VFS or 0.5mM sodium arsenite (ARS), and the presence of PG/SG analysed by live fluorescence imaging using GFP as PG/SG markers. **(D)** Following removal of the stressors and wash with PBS buffer, cells were left to recover, and images were taken every 10 minutes for 180 minutes (ARS)**,** or 180 minutes (VFS). Representative images are shown. **(E)** Fluorescence recovery after photobleaching (FRAP) analysis of GFP-G3BP1 granules in U2OS GFP-G3BP1 cells treated with 0.5 mM ARS or VFS. Plot shows recovery curves as an average of 9 granules for VFS (red) and 7 granules for ARS (green) ± SEM. Images were taken every 3s during recovery. Mean intensity was determined at each time point using ImageJ (It). Mean intensity of GFP within the cell from a different part of the cytoplasm was measured (IBt) to correct for bleaching during image acquisition. Corrected mean intensity at each time point was determined by taking the ratio: It/IBt and used to calculate the diffusion mobility coefficient of GFP-G3BP1.

Journal of Cell Science • Supplementary information

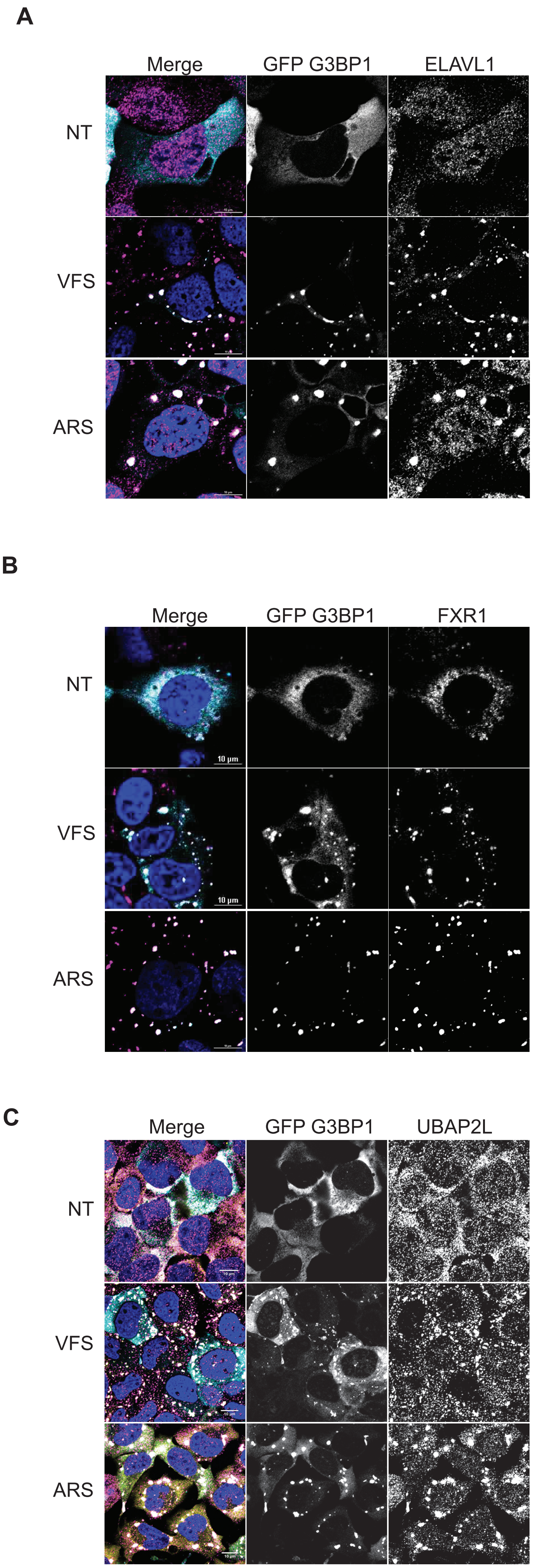
A
Merge
GFP G3BP1
ELAVL1
NT
VFS
ARS
B
Merge
GFP G3BP1
FXR1
NT
VFS
ARS
10 µm
10 µm
C
Merge
GFP G3BP1
UBAP2L
NT
VFS
ARS

Fig. S2. Validation of the PGs proteomic analysis and proteins shared with SGs by confocal microscopy. U2OS GFP-G3BP1 cells were stimulated for 1h with VFS or 0.5mM sodium arsenite (ARS) prior to fixation and the formation of PGs/SGs analysed by confocal microscopy. Non-treated (NT) were used as controls. Cells were stained with GFP (cyan) and ELAVL1 (magenta, **A)** or FXR1 magenta, **B)** or UBAP2L **(**magenta, **C)** as SG markers. Nuclei were stained with DAPI (blue). Scale bars, 10µm.

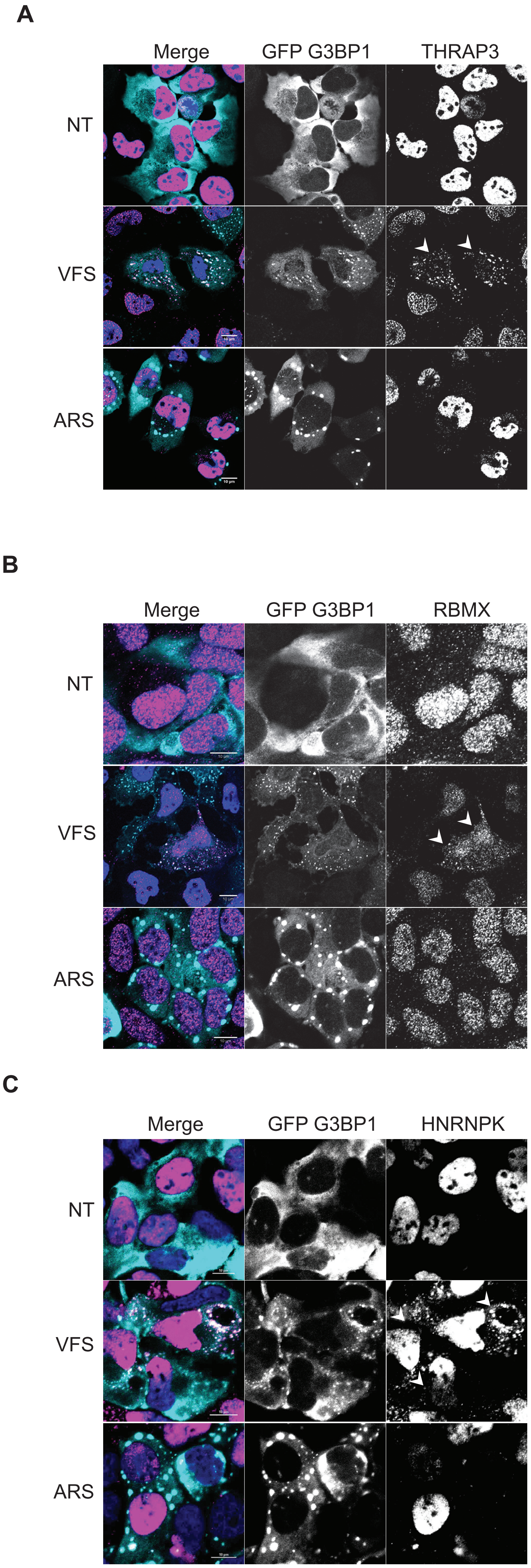

Journal of Cell Science • Supplementary information

Fig. S3. Validation of the PGs proteomic analysis and PG-specific proteins by confocal microscopy. U2OS GFP-G3BP1 cells were stimulated for 1h with VFS or 0.5mM sodium arsenite (ARS) prior to fixation and the formation of PGs/SGs analysed by confocal microscopy. Non-treated (NT) were used as controls. Cells were stained with GFP (cyan) and THRAP3 (magenta, **A)** or RBMX **(**magenta, **B)** or HNRNPK (magenta, **C)** as PG markers. Nuclei were stained with DAPI (blue). Cells with PGs are indicated with white arrows. Scale bars, 10µm.

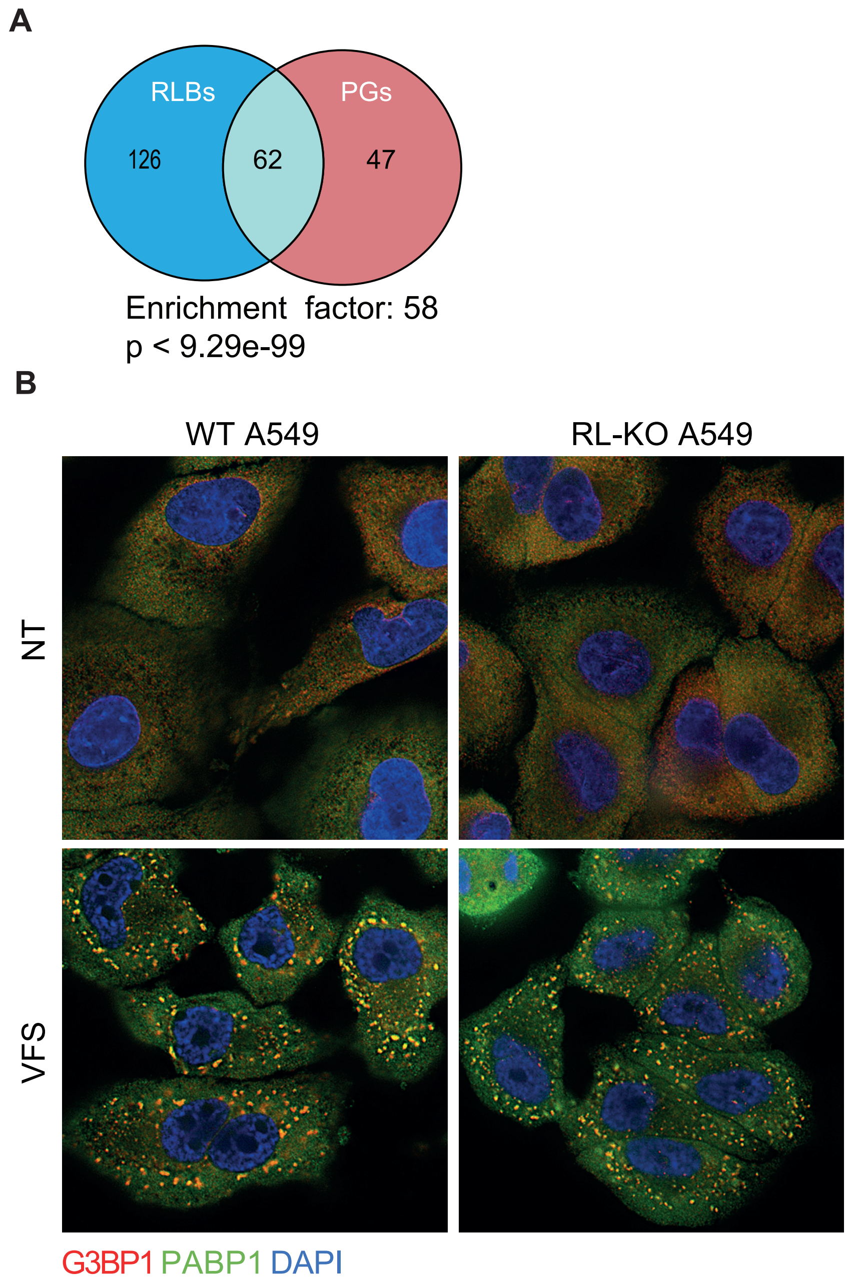

Fig. S4. Proteomic and immunofluorescence analysis reveals differences between VFS-induced PGs and RLBs. (A) Venn diagram between 110 proteins identified in PGs and 317 in RLBs (from (Burke et al., 2020)). The representation factor showed the enrichment over the expected and p-value is based on the cumulative distribution function (CDF) of the hypergeometric distribution of the data set over the mouse proteome. **(B)** Immunofluorescence analysis of PGs assembly in parental (WT) and RNase L–KO (RL-KO) A549 cells stained for G3BP1 and PABP1. Scale bars 10 μM.

Journal of Cell Science • Supplementary information

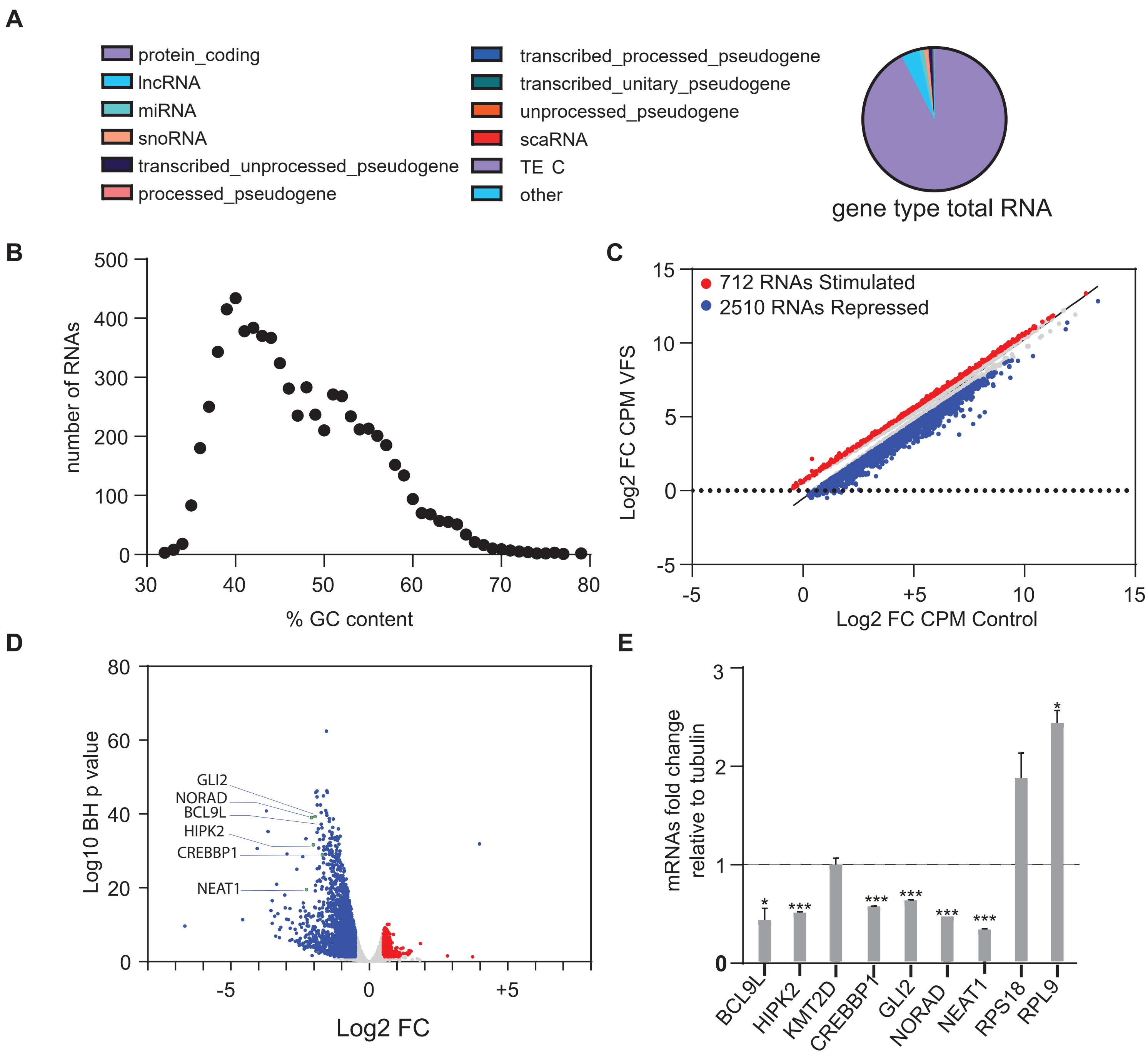

Fig. S5. VFS stimulation induces global changes in the transcriptome. (A) Pie chart analysis of the differentially expressed RNA identified by total transcriptome analysis of VFS-stimulated cells, compared to untreated cells, and clustered by RNA categories. **(B)** Scatterplot representation of GC content distribution across the RNA identified in **(A). (C)** Scatterplot of the Log2 CPM (count per million) for differential expression total RNAs between treated and/not with VFS, with analysis based on the R bioconductor package EdgeR. Differential expression was identified using GLM likelihood ratio tests. The blue dots indicate the significant RNAs with Log2 CPM< 0.5 and the red dots are the RNAs with Log2 CPM > 0.5, in grey the RNAs not significant (p-value>0.05) **(D)** Volcano plot of differential gene expression of U2OS cells stimulated with VFS versus non-treated controls. Each point represents the average value of one transcript in three replicate experiments. **(E)** Transcript levels of the paracrine induction were quantified via RT-qPCR relative to untreated and normalized to tubulin mRNA. Error bars represent SEM, n = 3. *P < 0.05, ***P < 0.005.

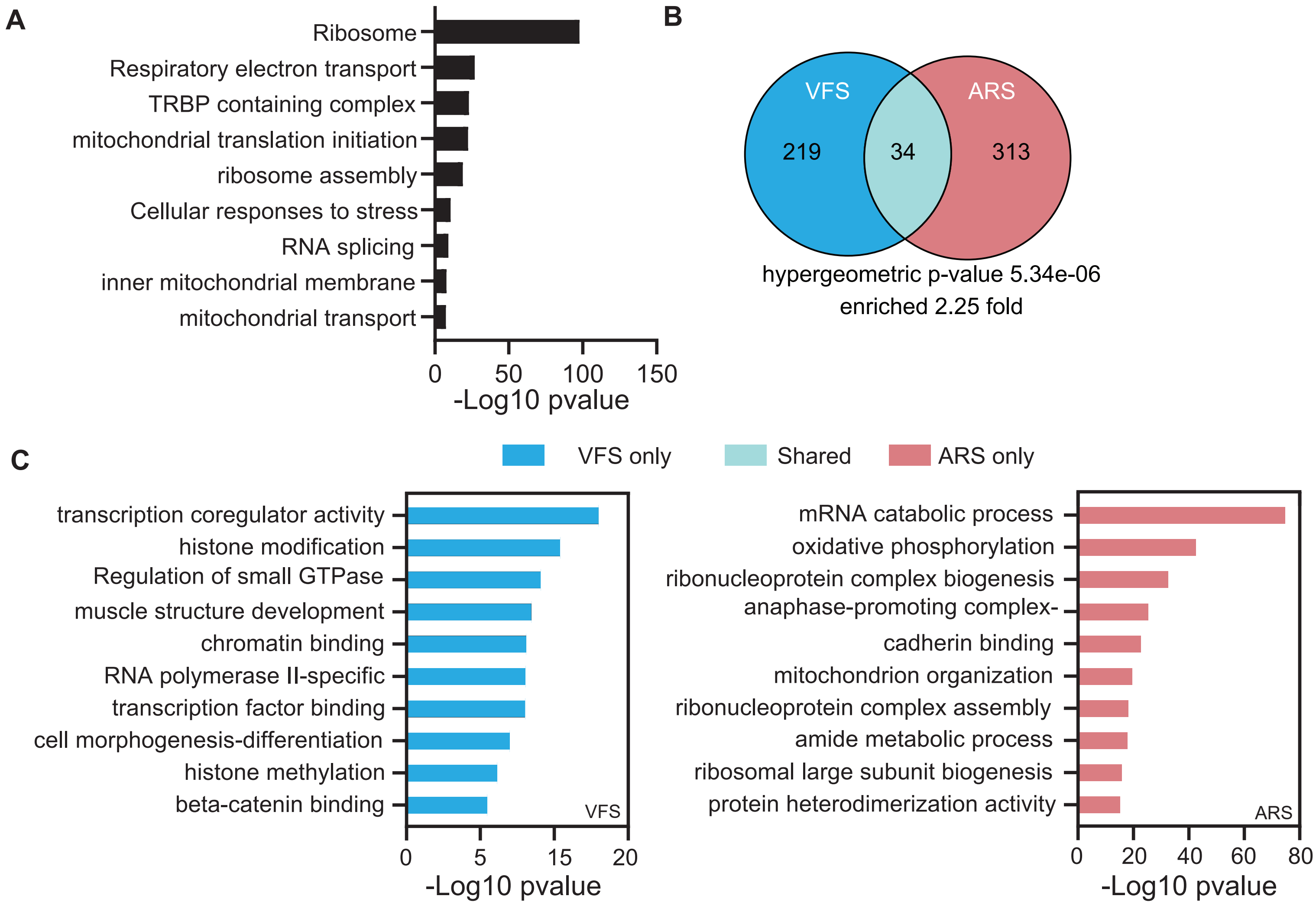

Fig. S6. Functional clustering reveals differences in transcriptome changes between VFS-treatment and oxidative stress in U2OS cells. (A) Clustering by functional pathways, identified from gene ontology analysis (KEGG/Reactome) of the differentially expressed RNAs in VFS-treated versus non-treated U2OS cells. **(B)**. Venn diagram comparison of the GO terms (molecular function and biological process) enriched in the differentially expressed U2OS transcripts upon VFS-stimulation or sodium arsenite (ARS) treatment (from (Khong et al., 2017)). **(C)** Comparison of the top 10 GO terms enrichment (molecular function and biological process) for the top 500 mRNAs differentially expressed following VFS (dark blue) or ARS (pink) stimulation of U2OS cells. GO terms overlapping both conditions are in light blue.

Journal of Cell Science • Supplementary information

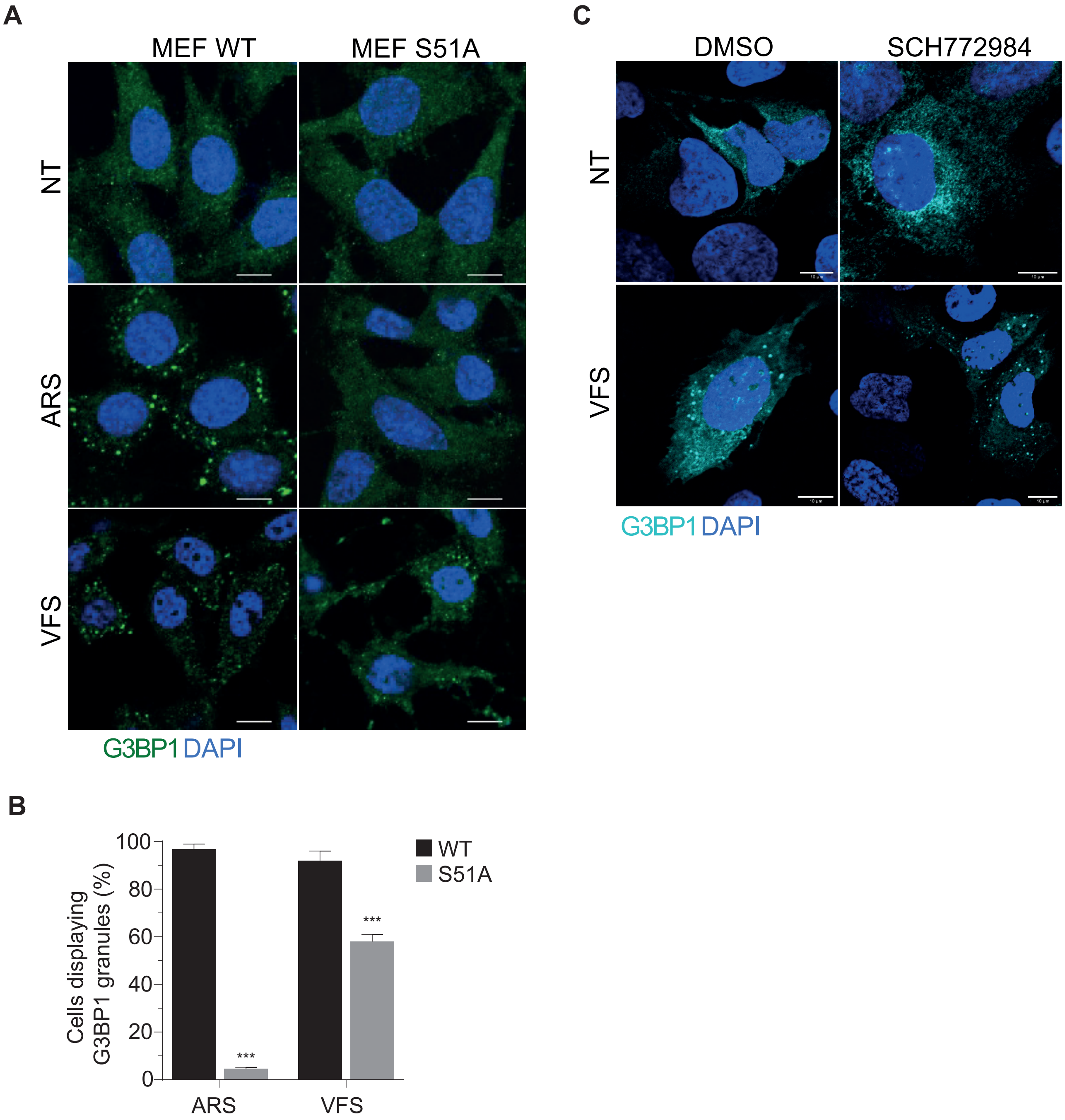

Fig. S7. ERK and eIF2α signalling are not essential for PG assembly. (A) WT or eIF2α S51A MEFs were stimulated for 1h with VFS or 0.5mM sodium arsenite (ARS) prior to fixation and the formation of stress granules analysed by confocal microscopy using G3BP1 as PG/SG markers. Non-treated (NT) cells were used as controls. Nuclei were stained with DAPI (blue). Scale bar is 10 μM. Representative bar plot (n=3) of the average number of cells displaying G3BP1 granules per cells is shown in **(B)**, with the mean ± SD for 100 G3BP1-positive cells analysed across at least 10 acquisitions, n = 3. ***P < 0.005. **(C)** U2OS cells were stimulated for 1h with VFS, with or without pre-treatment with the ERK inhibitor SCH772984 prior to fixation and the formation of stress granules analysed by confocal microscopy using G3BP1 as PG markers. Non-treated (NT) cells were used as controls. Nuclei were stained with DAPI (blue). Scale bar is 10 μM.

Journal of Cell Science • Supplementary information

Table S1. Proteins identified by Mass Spectrometry as PG residents.

Click here to download Table S1

Table S2. Comparison of proteins identified by Mass Spectrometry as PG residents with proteins previously identified as SG, PBs and PSPs components.

Click here to download Table S2

Table S3. Comparison of proteins identified by Mass Spectrometry as PG or arsenite-induced SG components.

Click here to download Table S3

Table S4. GO analysis of proteins identified by Mass Spectrometry as PG components.

Click here to download Table S4

Table S5. Total transcriptome analysis by RNAseq following VFS stimulation.

Click here to download Table S5

Table S6. Differential expression analysis of the transcriptome following VFS stimulation.

Click here to download Table S6

Journal of Cell Science • Supplementary information

Table S7. Comparison of GO terms enrichment for the top 500 significant RNAs identified in VFS or arsenite-treated U2OS cells.

Click here to download Table S7

Table S8. Stress granule transcriptome analysis following VFS stimulation.

Click here to download Table S8

Table S9. Comparison of GO terms enrichment for RNAs identified as PG or arsenite-induced SG components.

Click here to download Table S9

Table S10. Comparison of top 10 GO terms enrichment for RNAs identified as PG or arsenite-induced SG components.

Click here to download Table S10

Table S11. Comparison of top 10 enriched pathways for RNAs identified as PG or arsenite-induced SG components.

Click here to download Table S11

Journal of Cell Science • Supplementary information

Table S12. Antibodies used for immunoblotting and confocal microscopy

Antibody	Company	Catalogue number	Dilution	WB/IF
HRP-coupled goat anti-mouse	Dako	P0447	1:1000 to 1:5000	WB
HRP-coupled goat anti-rabbit	Dako	P0448	1:1000 to 1:5000	WB
eIF2alpha	Cell Signaling Technology	#9722	1:1000	WB
eIF2alpha Ser51	Cell Signaling Technology	#9721	1:1000	WB
eIF3b	Santa Cruz	Sc-16377	1:600	IF
eIF4E	Cell signaling	#9742	1:1000	WB
p38	Cell Signaling Technology	#9212	1:1000	WB
p38 T180/Y182	Cell Signaling Technology	#9211	1:1000	WB
mTOR	Cell Signaling Technology	#9283	1:1000	WB
mTOR Ser2448	Cell Signaling Technology	#5536	1:1000	WB
ERK1/2	Cell Signaling Technology	#9102	1:1000	WB
ERK1/2 Thr202/Tyr204	Cell Signaling Technology	#9101	1:1000	WB
AKT	Cell Signaling Technology	#9272	1:1000	WB
AKT S473	Cell Signaling Technology	#4060	1:1000	WB
ELAVL1	Santa Cruz	Sc-5261	1:2000<br>1:300	WB<br>IF
eIF3b	Santa Cruz	Sc-16377	1:600	IF
G3BP1	BD bioscience	#611127	1:1000<br>1:600	WB<br>IF
HNRNPK	Protein tech	#11426-1-AP	1:600	IF
DSG1	Protein tech	#24587-1-AP	1:600	IF
ERH	Protein tech	#15974-1-AP	1:600	IF
FUS	Protein tech	#11570-1-AP	1:600	IF
SFRS7	Novus bio	NBP1-92382	1:300	IF
FXR1	Merk Millipore	#05-1529	1:600	IF
FXR1	Novus Bio	NBP1-89546	1:600	IF
RBMX	Sigma	HPA057707	1:600	IF
THRAP3	Protein tech	#19744-1-AP	1:600	IF
RNPS1	Protein tech	#10555-1-AP	1:600	IF

NOLC1	Protein tech	#11815-1-AP	1:600<br>1:1000	IF<br>WB
YTHDF3	Protein tech	#25537-1-AP	1:600<br>1:1000	IF<br>WB
UBAP2L	Abcam	AB138309	1:300	IF
PABP	Abcam	AB21060	1:1000	IF
B23	SIGMA	B0556	1:600	IF
N6A Methyladenosine	Merk	ABE572	1:300	IF
Lamin B	Protein tech	#12987-1-AP	1:1000	WB
β-actin	Protein tech	#66009-1-AP	1:5000	WB
GAPDH	Santa Cruz	Sc-32233	1:20000	WB
Tubulin	Santa Cruz	Sc-5546	1:2000	WB
NS7	Described in (Humoud et al., 2016)		1:2000	WB
Alexa-Fluor 555 anti-rabbit	Invitrogen	A21428	1:600	IF
Alexa-Fluor 555 anti-mouse	Invitrogen	A21424	1:600	IF
Alexa-Fluor 555 anti-goat	Invitrogen	A21432	1:600	IF
Alexa-Fluor 647 anti-mouse	Invitrogen	A21463	1:600	IF

Table S13. Primers used for qPCR analysis

gene	forward	reverse
RpL9	5'-GGTGGGGTAACAGAAAGGAAC-3'	5'-CGTTGATGGGGAAGTGAGC-3'
RpS19	5'-CACGATGCCTGGAGTTACTG-3'	5'-CCAGCTTGACGGTATCCAC-3'
RpS18	5`-CCAAGAGGGCGGGAGAAC-3	5`-TATTTTCCATCCTTTACATCCTTCTG-3`
NORAD	5'-GTCCTGACGACAACGGACAA-3'	5'- TAGAATGAAGACCAACCGCCC-3'
NEAT1	5`-AGTTAAGGCGCCATCCTCAC-3	5`-CGTTGGTCAATGTTGTCCCC-3`
β-Actin	5'-CATTGGCAATGAGCGGTTC-3'	5'-CCACGTCACACTTCATGATGG-3'
β-Tubulin	5'-CTGAACCACCTTGTCTCAGC-3'	5'- AGCCAGGCATAAAGAAATGG-3'
AHNAK	5'- TACCCTTCCTAAGGCTGACATT-3'	5'- TTGGACCCTTGAGTTTTGCAT-3'
CREBBP	5'- CCTGCCACGTCACAGACTG-3'	5'- GGCCAGAGTTACTATTGAGGAGG-3'
KMT2D	5'- ACCTGGGAATGACTCTAAGATGT-3'	5'- CACGCCTTGCACTTCCAAGA-3'
BCL9L	5'- TGGATTCAGAGGCCAAAGAG-3'	5'- CCCACTGTACGGCTGCTT-3'
GLI2	5'- CCCCTACCGATTGACATGCG-3'	5'- GAAAGCCGGATCAAGGAGATG-3'
HIPK2	5'- CCCGTGTACGAAGGTATGGC-3'	5'- AGTTGGAACTCGGCTCTATTTTC-3'

Journal of Cell Science • Supplementary information
