# Supplementary material for: Novel stress granule-like structures are induced via a paracrine mechanism during viral infection
Source: J Cell Sci. 2022 Mar 2;135(4):jcs259194. doi: 10.1242/jcs.259194 (PMC8976915; doi:10.1242/jcs.259194)
Supplement: Reviewer comments [file joces-135-259194_review_history.pdf]

Novel stress granule-like structures are induced via a paracrine mechanism during viral infection

Valentina Iadevaia, James M. Burke, Lucy Eke, Carla Moller-Levet, Roy Parker and Nicolas Locker
DOI: 10.1242/jcs.259194

Editor: Maria Carmo-Fonseca

Review timeline

Original submission:	9 August 2021
Editorial decision:	13 September 2021
First revision received:	2 December 2021
Editorial decision:	10 January 2022
Second revision received:	11 January 2022
Accepted:	13 January 2022

Original submission

First decision letter

MS ID#: JOCES/2021/259194

MS TITLE: Paracrine granules are cytoplasmic RNP granules distinct from stress granules assembling in response to viral infection.

AUTHORS: Valentina Iadevaia, James M Burke, Lucy Eke, Carla Moller-Levet, Roy Robert Parker, and Nicolas Locker
ARTICLE TYPE: Research Article

We have now reached a decision on the above manuscript.

To see the reviewers' reports and a copy of this decision letter, please go to: https://submit-jcs.biologists.org and click on the 'Manuscripts with Decisions' queue in the Author Area. (Corresponding author only has access to reviews.)

As you will see, all three expert reviewers thought that the work was potentially quite interesting and significant but all also raised a number of concerns that must be dealt with before the manuscript can be reconsidered. Please address these issues as thoroughly as possible. If you think that you can deal satisfactorily with the criticisms on revision, I would be pleased to see a revised manuscript. We would then return it to the reviewers. Note that no further experiments are required.

We are aware that you may be experiencing disruption to the normal running of your lab that makes experimental revisions challenging. If it would be helpful, we encourage you to contact us to discuss your revision in greater detail. Please send us a point-by-point response indicating where you are able to address concerns raised (either experimentally or by changes to the text) and where you will not be able to do so within the normal timeframe of a revision. We will then provide further guidance. Please also note that we are happy to extend revision timeframes as necessary.

© 2022. Published by The Company of Biologists under the terms of the Creative Commons Attribution License (https://creativecommons.org/licenses/by/4.0/).

Please ensure that you clearly highlight all changes made in the revised manuscript. Please avoid using 'Tracked changes' in Word files as these are lost in PDF conversion.

I should be grateful if you would also provide a point-by-point response detailing how you have dealt with the points raised by the reviewers in the 'Response to Reviewers' box. Please attend to all of the reviewers' comments. If you do not agree with any of their criticisms or suggestions please explain clearly why this is so.

Reviewer 1

Advance summary and potential significance to field

Iadevaia at al describe a novel type of RNP granule forming in cells subjected to virus-free cell lysates, and hence induced in the absence of active viral particles, suggesting a paracrine mechanism of induction. These granules share several properties and multiple protein/RNA components with canonical stress granules (such as those induced by arsenite), yet they were found to display a number of unique characteristics, for example cychoheximide insensitivity and lack of G3BP1 dependence. Reports of such a route of RNP granule induction are very scarce in the literature therefore the study is important and timely. It is also a thorough, well-executed study that utilised a variety of approaches from granule kinetics analysis to granule purification coupled with compositional analysis, to their antiviral activity. The manuscript will benefit from some restructuring, and more convincing and illustrative images/panels should be also provided in support of some data.

Comments for the author

Specific comments:
1. Introduction, last paragraph: three features distinguishing PGs from SGs are listed, with feature #3 being its antiviral activity. However, SGs are also antiviral therefore this feature cannot be listed as PG-specific.
2. It would be logical to transfer the data from the “G3BP1 is not essential for PGs assembly” subheading to the beginning of the manuscript as this data is related to initial (morphological) characterisation of PGs - this subheading looks out of place where it is now and disrupts the narrative. This way, several protein markers, not only FMR1, can be shown in Fig 1F, including UBAP2L (it would be helpful to add a couple of other classic markers eg TIA1 TIAR).
3. Fig 1G legend: “For forced SG disassembly, cells were treated with 10 µg/ml of CHX for 30min (+CHX)” – it is essential to indicate that the cells were pre-treated. Was CHX left in the media during stress as well? This information must be in the legend. In the VFS + CHX image, PGs look larger than in the VFS – CHX one, is it indeed the case or the image is not representative?
4. In Fig 1C and G, it should be indicated on the panel which protein marker was used.
5. Fig 1 D and E: “Representative bar plot (n=3) of the percentage of U2OS cells displaying G3BP1 foci” – this description is confusing: what is N here? “Representative” should be removed.
6. Fig. 2B: Legend states that “Dynabeads bound to GFP analysed by epifluoresence microscopy in ARS or VFS treated U2OS GFP-G3BP1 cells” however images only show NT (non-treated?) and VFS-treated cells but not ARS-treated cells. More importantly, beads after pull-down from NT cells and VFS-treated cells look identical in terms of protein capture but VFS beads are larger, why is this the case? In NT cells, I also see granule-like structures which although not indicated with arrows, look similar to the ones in the VFS image.
This panel needs to be corrected to provide convincing data. Finally, ‘G3BP1-GFP dynabeads’ title is inaccurate, these beads should contain SG cores and not diffuse G3BP1-GFP removed during washes, as can be implied.
7. Fig. 4B and D. The individual panels for the markers should be provided as it is impossible to clearly judge the distribution of each marker from the merged images. In B, the images unfortunately do not serve their purpose - the grainy pattern of m6A looks same in all three conditions and co-localisation with granules is not obvious. Magnified images or insets are needed to demonstrate co-localisation. In D, it looks like CHX has an effect on PGs (unlike in Fig 1 images) – granules look smaller with CHX. Scale bars are missing from both panels.
8. Page 9: “G3BP1 is not essential for PGs assembly” subheading. An obvious question here - is UBAPL2 essential for PG assembly?

© 2022. Published by The Company of Biologists under the terms of the Creative Commons Attribution License (https://creativecommons.org/licenses/by/4.0/).

9. Fig. 6A: In the images shown, FMR1 signal is drastically reduced in the arsenite and VFS treated cells – to the same extent as puromycin signal (more in asrenite treated cells) which is odd.
10. Fig. 6C: eIF2a total levels appear significantly downregulated upon stress (arsenite or VFS; actin appears similar in the three conditions). Is it a consistent finding in authors' experiments, how does it link to the existing literature and what can cause it?
11. Page 10: "PGs playing an antiviral response" subheading – "a role in" missing in the middle?
12. Fig. 7. A strong reduction in viral replication without induction of the major antiviral factors appears striking to me. Since PGs form rapidly suggesting that the cellular response to VFS treatment is swift, it is plausible that antiviral transcript levels ramp up fairly quickly too during treatment too. Have the transcripts been measured also relative to basal levels rather than comparing the 1 h and 6 h time-points?
13. Manuscript is in need of careful proof-reading to correct multiple typos, word omissions and grammar issues, e.g.: "trigged by the infection" in abstract; "First, their assembly-disassembly pattern are different.." in introduction; "the assembly of PGs is a prosurvival events.." in introduction;
"kinetic of assembly-disassembling" in results – is it "kinetics of assembly-disassembly"; "potential reflecting" in results; "we could not measure any difference.." in results – could not measure or detect?; "took advantage an affinity-based SG isolation.." in results; "we observed an increased in proteins" in results; "differentially expression analysis" in results;
"process and unprocessed pseudogenes" in results, "cores can also assembly around" in results, etc.

Minor
1. I am not entirely sure about the term "paracrine granules", as it somewhat diminishes the significance of the authors' discovery. Also, "paracrine" refers to the mechanism of their assembly rather than their nature or location, unlike for all other types of RNA/RNP granules we know. Perhaps at least in the title, these granules should be referred to as "Novel stress granule-like structures induced via a paracrine mechanism in viral infections".
2. Abstract: "G3BP1-granules" – not clear what is meant here – G3BP1-positive granules?
3. Page 8: "Therefore, we compared the GO terms enrichment for both SGs and PGs RNAs and for the SGs induced by arsenite in U2OS GFP-G3BP1 cells (Fig. 3F). This revealed a significant overlap between the two classes of condensates (16.3 fold).." – not clear how overlap can be 16-fold – compared to what?
4. Page 9: "To ascertain whether PGs and RLBs are in fact similar aggregates we compared their proteins composition." – was not the goal here to confirm that RLBs and PGs are different, not similar or identical, structures?
5. "G3BP1 interactors and SG cores were then enriched by sequential centrifugation.." – this is confusing – SG cores were purified and as a result G3BP1 interactors established.
6. The Summary statement is too generic, it needs to be revised to report the key features of the new granule type described in the study.

<u>Reviewer 2</u>

Advance summary and potential significance to field

The manuscript expands on an exciting observation where following infection with FCV, SGs are induced in the neighboring non-infected cells. The authors reproduce this results using the media collected from infected cells, and late on in the paper show that pre-treatment with this media protects the cells against subsequent challenge with virus. In order to understand these observations, the authors undertake a careful and comprehensive characterization of the granules in terms of composition and dynamics of formation. They also start to dissect some of the signalling pathways that may be involved. I found the paper intriguing and novel, but do have some relatively minor points for the author's attention.

Comments for the author

1. The authors are keen on the idea that a metabolite or protein from the virally infected cells induces granule formation in the neighboring cells. Can they rule out the absence of a

© 2022. Published by The Company of Biologists under the terms of the Creative Commons Attribution License (https://creativecommons.org/licenses/by/4.0/).

component in the media e.g. a key nutrient such as glucose or amino acids- where the absence might induce a form of stress. Have they tried supplementing VFS with key nutritional components and assessing whether the SGs still form?
2. Cycloheximide is thought to prevent the formation of stress granule because it traps mRNA on polysomes. Cycloheximide does not prevent accumulation of granules described in this paper. If mRNA is trapped on polysomes, then are the polysomes associated with the granules i.e. are the granules associated with active translation- especially since there is no real stress here! Could they be the translation factories that have recently been suggested in multiple systems?
3. I feel the authors could go further in their conclusion to the FRAP experiments. They show that the dynamics of G3BP1 are similar between arsenite and VFS treatment, and G3BP1 is a known requirement for arsenite stress granules- yet the kinetics of formation and disintegration for the two granule types are different. This could be used to lead into the experiments using cell lines where G3BP1 is deleted.
4. I felt the authors could have commented further on the proteins present in the granules. One view of stress granules is that they contain stalled ribosomal preinitiation complexes. From the proteomics, there are several ribosomal subunits both 40S and 60S, but only one initiation factor eIF5B, which is associated with a late subunit joining step of the process. Intriguingly there are no eIF3 subunits- this supports the authors view that these granules are distinct from arsenite induced stress granules (in fact many would argue the new granules are not stress granules on this basis).
5. In Figure 7A B, the authors measure the impact of FCV on puromycin incorporation. They should also address whether initiation is specifically affected by assessing polysome run-off.
6. In Figure 7C, the authors address potential signaling alterations that correlate with granule formation. The increase in eIF2alpha P is very minimal and requires statistical support if the authors are going to interpret as they currently do. The most convincing change to my mind is the increase in ERK phosphorylation. My understanding is that such an increase is often associated with serum stimulation and increased translation. So this experiment raises many intriguing questions that could easily be addressed by expanding the antibodies used and or the use of some inhibitors. So does S6 phosphorylation increase? The authors state it doesn't - but I can't find the data here? What about the S6 kinases are these activated? Past literature claims there are connections between stress granules and different S6 kinases (e.g. Eisinger-Mathason et al., 2008. Mol Cell; Sfakianos et al., 2018. Cell Death Diff.). Other past literature connects mild stress with stress granule formation and ERK signaling (e.g. Meyerowitz et al., 2011. Mol Neurodegeneration). So could they use inhibitors of JNK and ERK? With some minimal extra work here, the authors may be able to provide a more concrete signalling mechanism for the formation of these granules.
7. The last sentence in the results states 'These suggests that the VFS-induced reduction in viral replication may be due to translational stalling rather than activation of antiviral signalling in cells assembling PGs'. My understanding is that the authors haven't done any experiments to address translational stalling- so this statement needs to be toned down- unless the polysome experiment suggested above gives evidence for translation stalling.

Manuscript presentation comments:
1. In Figure 1F, the G3BP1 panels are very faint and difficult to see- the authors should consider replacing this panel with another image or increasing the brightness/contrast so that the G3BP1 signal is more evident.
2. For the time lapse microscopy and other images across the paper- Because the granules following VFS treatment are smaller than the stress granules formed after arsenite treatment, they are more difficult to see. A zoomed in panel for many of the images would help to overcome this problem.
3. The order of the paper seemed odd in places – as alluded to above the FRAP data may be better with the investigation of G3BP1 requirement. Plus where the FRAP data is presented in Figure 1H- a quantitative assessment of the differences observed in dynamics from the time course might be more interesting.

4. The final figure deals with the question most general readers are probably asking from the outset- that is does the process where these granules are induced in cells that neighbour infected cells alter their capacity for infection. I would move this to after Fig 1. – so the paper flow would be – 1. The granules form and appear different to stress granules. 2. They are part of a protection mechanism. 3. Their composition and regulation

© 2022. Published by The Company of Biologists under the terms of the Creative Commons Attribution License (https://creativecommons.org/licenses/by/4.0/).

<u>Reviewer 3</u>

Advance summary and potential significance to field

Iadevaia et al.
Paracrine granules are cytoplasmic RNP granules distinct from stress granules that assemble in response to viral infection

The manuscript by Iadevaia et al. has exciting potential as the authors have attempted to tackle two fundamentally important questions related to RNA granule biology and virology. 1) Are all stress granules (SGs) equal, and if not, what is the functional significance of distinct subsets of SG-like aggregates? 2) Are SGs or related granules like those they identify here part of a cellular antiviral response designed to restrict viral replication?

The authors describe a novel ribonucleoprotein granule associated with virus infection that was initially observed in bystander uninfected cells in cell monolayers infected with feline calicivirus (FCV). Because these granules form in response to paracrine factors released from infected cells, the authors have named them paracrine granules (PGs). The authors characterize PGs and show they are distinct from stress granules (SGs) and other known ribonucleoprotein granules in terms of their kinetics of assembly and disassembly, RNA and protein composition. The authors complete a comprehensive proteomic and transcriptomic analysis of isolated PGs, capitalizing on the method previously used by Jain et al. to characterize SGs. I commend the authors for the incredible data set provided in this manuscript.

Despite this, I remain unconvinced that the granules that have been characterized in such detail are indeed the same granules in terms of composition as those first identified in uninfected bystander cells in FCV-infected monolayers. I have two major reasons for this concern.

Comments for the author

Major Concerns to be addressed:

1. A very interesting feature of the data that is not highlighted enough by the authors is that UV-inactivated virus (Fig 1A) does not induce PGs yet virus-free supernatant (VFS) does (Fig 1C). It is difficult to understand why this is. At minimum, the authors should provide more information as to the difference between these preparations. For example, UV is used to inactivate any viral particles that remain in the VFS after PEG-precipitation – presumably prior to adding this inactivation step, there was some residual infectivity? Could the authors perform RT-qPCR to quantify viral genomic RNA prior to UV treatment in VFS compared to the viral preps used in Fig 1A? This would quantify the number of inactivated viral particles that are found in each preparation, and help determine if any of the effects observed result from virus particle entry. Moreover, could the authors try other strategies to better characterize the VFS? DNase? RNase? Proteases treatments? Transwell assays? This could help identify the nature of the molecule(s) that induces the PGs, a central question for any paracrine effect.
As the premise of this work is to link these novel granules to IFN or antiviral responses, could the authors examine if the granules form in response to treatment with TypeI IFNs? Or could there be another way to induce PGs that does not rely on a supernatant of an unidentified nature? Perhaps induction of cytoplasmic crowding and LLPS, or overexpression of PG-unique proteins, or a combination of these approaches?
Finally, it is important to show the TCID50 assays that were used to verify the absence of infectious viral particles from the VFS and are referred to as data not shown. The preparation and the nature of the VFS is important to understand as it the treatment upon which all subsequent experiments are based.
2. A central concern with the study is the circular argument regarding G3BP1. The identification of PGs and most of the analysis shown in this manuscript relied on overexpression of gfp-G3BP1 or immunofluorescence for endogenous G3BP1. The isolation of PGs for extensive proteomic and RNA analysis also relied on overexpressed of gfp-G3BP1, and the pulldown was performed using a gfp antibody, therefore, all the subsequent analysis of the protein and RNA components of PGs has been performed on granules that contain G3BP1.

© 2022. Published by The Company of Biologists under the terms of the Creative Commons Attribution License (https://creativecommons.org/licenses/by/4.0/).

Fig5 shows data that is intended to validate the formation of PGs using antibodies to proteins that are common to PGs and SGs (Fig 5A) and unique to PGs (Fig 5B). In Fig 5B, the panels indicate that cytoplasmic puncta form that are THRAP3 and RBMX positive after VFS treatment and these puncta co-stain with GFP-positive puncta in cells that are overexpressing gfp-G3BP1. There are no cytoplasmic puncta that stain positive for PG-unique marker proteins THRAP3 and RBMX in any cells that do not overexpress gfp-G3BP1. From the data shown, I can only infer that G3BP1 is a required scaffold or catalyst for PG formation.
The third panel intended to analyze the PG-unique protein hnRNPK is impossible to decipher because the immunofluorescent staining is of poor quality.
These data become more concerning when placed in context with the data shown in Fig S6, wherein the authors state that PGs form in the absence of G3BP1. In these images, cytoplasmic puncta are present in VFS-treated cells in the absence of G3BP1 but these are visualized by staining for FXR1 and UBAP2L – proteins that the authors state are common to PGs and SGs. Therefore, at no time in the manuscript do the authors validate the formation of PGs with PG-specific protein markers like THRAP3 and RBMX in the absence of G3BP1. So that makes me wonder – what did they isolate? Is the granule that is characterized the same granule that is visualized?
I find the inconsistency with these data troubling. Hopefully the authors have additional data that is not yet in the manuscript that can be added to show that PGs form in response to VFS and in the absence of G3BP1 and that these granules can be stained with markers that are unique to PGs, as identified by the authors.

Minor Concerns:

1. The major premise of the work is that PGs may be a subtype of SGs that are antiviral. The authors do not highlight any IFN signaling molecules found in these granules, and inflammatory cytokines are not altered in cells treated with VFS. In the final figure of the paper, the authors show that VFS pre-treatment limits viral replication, but no evidence is provided to show that the mechanism of this inhibition is anything other than translation shutoff. PGs, although enriched in signaling molecules/their transcripts, do not appear to function in antiviral signaling. This means that PGs do not have a novel function. Combined with the above concerns, I guess I am unclear how different they are from SGs.

2. I do not understand the disconnect between the more rapid assembly/disassembly properties of PGs relative to SGs (Fig S1) and the observations that FRAP was indistinguishable between PGs and SGs. Is this because G3BP was overexpressed for FRAP making the granule artificially stable and limiting exchange of contents? Please comment in the manuscript on this issue.

3. Several typos found.
-please add line numbers to better allow reference to the typos in the text such as:
-PKR listed twice as a signalling molecule recruited to SGs, both in the intro and in results sections
-'in response in response' – repeated phrase end of p9
-typo in results re Fig 1E - significant instead of significantly
-assembly-disassembling end of p4
-potential instead of potentially top of p5
-m in mitochondrial is cut off in Fig S5A
-typo in FigS6 legend – SGs should read RLBs I think

First revision

Author response to reviewers' comments

Dear Dr. Maria Carmo-Fonseca,

Thank you for your initial assessment of our study entitled 'Paracrine granules are cytoplasmic RNP granules distinct from stress granules assembling in response to viral infection' (JOCES/2021/259194) and for giving us the opportunity to revise our manuscript.

© 2022. Published by The Company of Biologists under the terms of the Creative Commons Attribution License (https://creativecommons.org/licenses/by/4.0/).

As you highlighted, and as suggested by reviewers #1, #2 and #3, we have revised, restructured our manuscript and provided clarifications/corrections where requested. We noted your comment that no further experimental work was required, yet we have provided additional data not present in the original submission when possible to help answer specific comments.

We do hope that you will find this improved version of our manuscript, now entitled ‘Novel stress granules-like structures are induced via a paracrine mechanism during viral infection’, suitable for publication in Journal of Cell Science.

Kind regards
Nicolas Locker

Specific comments to the Editor

As suggested, we have:
1- Moved the tables from Material and Methods into the supplementary PDF file.
2- Changed the title to comply with word limit and reviewer’s comments.
3- Reduced the abstract length and manuscript length.
4- Added missing funding information on the online submission form.

Specific comments to the Reviewers

Response to Reviewer #1:

We fully appreciate the comments made by this reviewer and took them onboard to improve our manuscript as outlined in our response to their comments.

Specific comments:

1.Introduction, last paragraph: three features distinguishing PGs from SGs are listed, with feature #3 being its antiviral activity. However, SGs are also antiviral therefore this feature cannot be listed as PG-specific.

The exact role of SGs during viral infection is still the subject of debate (as reviewed in Eiermann et al, Viruses 2020 and Mateju et al, FEBS Letters 2021) with antiviral granules, distinct from stress granules, clearly linked to antiviral signalling activation while SG components such as G3BP1 can regulate antiviral signalling in infected cells, independently from their SG localisation. It is unclear to us whether canonical SGs (i.e. induced by arsenite) are associated with antiviral activity, hence the statement made. However to reflect the reviewer we have amended the abstract to highlight that PGs are associated with antiviral activity (line 28) rather than contrast it to an SG property.

2.It would be logical to transfer the data from the “G3BP1 is not essential for PGs assembly” subheading to the beginning of the manuscript as this data is related to initial (morphological) characterisation of PGs - this subheading looks out of place where it is now and disrupts the narrative. This way, several protein markers, not only FMR1, can be shown in Fig 1F, including UBAP2L (it would be helpful to add a couple of other classic markers eg TIA1, TIAR).

We followed this suggestion from reviewer #1, and #2, and the data are now discussed line 160 onwards with the data from Figure 5 now presented as Figure 2. In addition, as suggested a new panel 1F (previously 5A) now shows the UBAP2L/FXR1/GFP triple staining of PGs/SGs.

3.Fig 1G legend: “For forced SG disassembly, cells were treated with 10μg/ml of CHX for 30min (+CHX)” – it is essential to indicate that the cells were pre-treated. Was CHX left in the media during stress as well? This information must be in the legend. In the VFS + CHX image, PGs look larger than in the VFS – CHX one, is it indeed the case or the image is not representative?

We have added a more representative image as suggested and amended the legend as follow: VFS-induced G3BP1 aggregates are CHX insensitive. U2OS GFP-G3BP1 cells were pre-treated with ARS or VFS and for forced SG disassembly, treated with 10μg/ml of CHX for the final 30min (+CHX). Of

© 2022. Published by The Company of Biologists under the terms of the Creative Commons Attribution License (https://creativecommons.org/licenses/by/4.0/).

note, we measured the average granule size and could not detect any difference +/- CHX (not shown).

4.In Fig 1C and G, it should be indicated on the panel which protein marker was used.

This has been added as suggested.

5.Fig 1 D and E: "Representative bar plot (n=3) of the percentage of U2OS cells displaying G3BP1 foci" – this description is confusing: what is N here? "Representative" should be removed.

Representative has been removed as suggested.

6.Fig. 2B: Legend states that "Dynabeads bound to GFP analysed by epifluoresence microscopy in ARS or VFS treated U2OS GFP-G3BP1 cells" however images only show NT (non-treated?) and VFS-treated cells but not ARS-treated cells. More importantly, beads after pull-down from NT cells and VFS-treated cells look identical in terms of protein capture but VFS beads are larger, why is this the case? In NT cells, I also see granule-like structures which, although not indicated with arrows, look similar to the ones in the VFS image. This panel needs to be corrected to provide convincing data. Finally, 'G3BP1-GFP dynabeads' title is inaccurate, these beads should contain SG cores and not diffuse G3BP1-GFP removed during washes, as can be implied.

This is now discussed in the manuscript, line 191 onwards. In brief, previous mass spectrometry have already shown that G3BP1 interactions pre-exist in unstressed cells as preformed RNPs but that these only condense into visible SG structures upon stress, thus explaining why most G3BP1 interactors are also found in unstressed conditions and in this case why background staining of the dynabeads occurs in NT cells, although without detection of GFP-G3BP1 foci. The title below has been changed to clear any confusion, the panel size adjusted to reflect identical bead size and more representative pictures added removing the unnecessary low/high exposure.

7.Fig. 4B and D. The individual panels for the markers should be provided as it is impossible to clearly judge the distribution of each marker from the merged images. In B, the images unfortunately do not serve their purpose - the grainy pattern of m6A looks same in all three conditions and co-localisation with granules is not obvious. Magnified images or insets are needed to demonstrate co-localisation. In D, it looks like CHX has an effect on PGs (unlike in Fig 1 images) – granules look smaller with CHX. Scale bars are missing from both panels.

This is now replaced by Figure 5. We agree that the quality of the m6A staining is not optimal, and this is something that others have reported as well. We have selected the panels and increased the resolution which hopefully conveys better the message. Panel D has been replaced to show the split channels and compare the NT and VFS conditions as displaying the +/- CHX was not essential for this part of the manuscript, especially given the CHX importance is already addressed in Figure 1.

8.Page 9: "G3BP1 is not essential for PGs assembly" subheading. An obvious question here - is UBAPL2 essential for PG assembly?

We investigated the requirement for UBAP2L in Figure 2, and while UBAP2L is not essential for PG assembly, its deletion results in a smaller number of PG/cells following stimulation with VFS (line 172, Figure 2B and 2C). Therefore, we propose that UBAP2L may contribute to the PG maturation process.

9.Fig. 6A: In the images shown, FMR1 signal is drastically reduced in the arsenite and VFS treated cells – to the same extent as puromycin signal (more in asrenite treated cells) which is odd.

Given that overall protein synthesis is reduced followed ARS and VFS treatment it is perhaps not surprising to observe reduced level of FMR1, which together with its localisation to distinct foci (SG or PG) would explain why the overall signal is reduced.

10.Fig. 6C: eIF2a total levels appear significantly downregulated upon stress (arsenite or VFS; actin appears similar in the three conditions). Is it a consistent finding in authors' experiments, how does it link to the existing literature and what can cause it?

© 2022. Published by The Company of Biologists under the terms of the Creative Commons Attribution License (https://creativecommons.org/licenses/by/4.0/).

We displayed this replicate for consistency with the other proteins analysed, to only display blots obtained from a single replicate. It had overall the best quality of images, yet we agree that eIF2a level look weaker however we did not observe this in other replicates, therefore did not follow up on this observation.

11.Page 10: “PGs playing an antiviral response” subheading – “a role in” missing in the middle?

Corrected in line 384.

12.Fig. 7. A strong reduction in viral replication without induction of the major antiviral factors appears striking to me. Since PGs form rapidly suggesting that the cellular response to VFS treatment is swift, it is plausible that antiviral transcript levels ramp up fairly quickly too during treatment too. Have the transcripts been measured also relative to basal levels rather than comparing the 1 h and 6 h time-points?

The transcript levels are normalised to the corresponding time points in control supernatant (i.e from uninfected cells) treated cells. Impact on viral replication could be explained in many ways which include for example affecting intracellular trafficking to block viral egress, impaired maturation of viral proteins, genome packaging and membrane re-arrangements, all of which would not directly results from ISGs activation.

13.Manuscript is in need of careful proof-reading to correct multiple typos, word omissions and grammar issues,e.g.: “trigged by the infection” in abstract; “First, their assembly-disassembly pattern are different..” in introduction; “the assembly of PGs is a prosurvival events..” in introduction; “kinetic of assembly-disassembling” in results – is it “kinetics of assembly-disassembly”; “potential reflecting” in results; “we could not measure any difference..” in results – could not measure or detect?; “took advantage an affinity-based SG isolation..” in results; “we observed an increased in proteins” in results; “differentially expression analysis” in results; “process and unprocessed pseudogenes” in results, “cores can also assembly around” in results, etc.

We wholeheartedly apologize for these errors and have corrected all the above in addition to careful proof-reading and editing of further typos.

Minor

1.I am not entirely sure about the term “paracrine granules”, as it somewhat diminishes the significance of the authors’ discovery. Also, “paracrine” refers to the mechanism of their assembly rather than their nature or location, unlike for all other types of RNA/RNP granules we know. Perhaps at least in the title, these granules should be referred to as “Novel stress granule-like structures induced via a paracrine mechanism in viral infections”.

We took the comments onboard and amended our title to ‘Novel stress granules-like structures are induced via a paracrine mechanism during viral infection’. However we maintained the paracrine granules domination for the novel SG-like foci we describe as we sum up best the key message of the paper.

2.Abstract: “G3BP1-granules” – not clear what is meant here – G3BP1-positive granules?

The abstract has been corrected G3BP1-positive granules, line 26.

3.Page 8: “Therefore, we compared the GO terms enrichment for both SGs and PGs RNAs and for the SGs induced by arsenite in U2OS GFP-G3BP1 cells (Fig. 3F). This revealed a significant overlap between the two classes of condensates (16.3 fold)..” – not clear how overlap can be 16-fold – compared to what?

Compared to the number of GO terms for molecular functions and biological processes published on Metascape.

4.Page 9: “To ascertain whether PGs and RLBs are in fact similar aggregates we compared their proteins composition.” – was not the goal here to confirm that RLBs and PGs are different, not similar or identical, structures?

© 2022. Published by The Company of Biologists under the terms of the Creative Commons Attribution License (https://creativecommons.org/licenses/by/4.0/).

This has now been rephrased, see line 221.

5.“G3BP1 interactors and SG cores were then enriched by sequential centrifugation..” – this is confusing – SG cores were purified and as a result G3BP1 interactors established.

This has now been rephrased, see line 185.

6.The Summary statement is too generic, it needs to be revised to report the key features of the new granule type described in the study.

The summary statement has been rewritten as suggested, see line 15.

Response to Reviewer #2:
1.The authors are keen on the idea that a metabolite or protein from the virally infected cells induces granule formation in the neighboring cells. Can they rule out the absence of a component in the media e.g. a key nutrient such as glucose or amino acids- where the absence might induce a form of stress. Have they tried supplementing VFS with key nutritional components and assessing whether the SGs still form?

To clarify, our control treatment corresponds to a virus-free supernatant generated from uninfected cells, collected and purified exactly like VFS. So media depletion due to cell growth shouldn’t alone explain our results. We do not want to restrict ourselves when seeking for the mediator identity but have performed preliminary experiments showing the VFS effect is heat dependent. We have generated a secretome MS dataset from cells infected with FCV and analysis is currently underway, this will form the basis of a further manuscript exploring in more details the paracrine effect and its mediator.

2.Cycloheximide is thought to prevent the formation of stress granule because it traps mRNA on polysomes. Cycloheximide does not prevent accumulation of granules described in this paper. If mRNA is trapped on polysomes, then are the polysomes associated with the granules i.e. are the granules associated with active translation- especially since there is no real stress here! Could they be the translation factories that have recently been suggested in multiple systems?

We thank the reviewer for this provocative thought. Our understanding is that translation factories are associated with localised translation of a subset of mRNAs to increase their local concentration and mediate the assembly of functional complexes. Herein we did not observe functional enrichment of specific classes of mRNAs when compared to SG resident proteins and thus we do not think PGs are in fact SGs. In addition, at the resolution used for this study we did not observed increased translation (puromycin signal) in PG foci but future experiments could be planned to investigate using single molecule approaches whether mRNAs localised to PGs are also translated in PGs.

3.I feel the authors could go further in their conclusion to the FRAP experiments. They show that the dynamics of G3BP1 are similar between arsenite and VFS treatment, and G3BP1 is a known requirement for arsenite stress granules- yet the kinetics of formation and disintegration for the two granule types are different. This could be used to lead into the experiments using cell lines where G3BP1 is deleted.

As suggested by the reviewer and reviewer #1 we have restructured the manuscript with the FRAP experiment leading into G3BP1 requirement for PG assembly (line 160).

4.I felt the authors could have commented further on the proteins present in the granules. One view of stress granules is that they contain stalled ribosomal preinitiation complexes. From the proteomics, there are several ribosomal subunits both 40S and 60S, but only one initiation factor eIF5B, which is associated with a late subunit joining step of the process. Intriguingly there are no eIF3 subunits- this supports the authors view that these granules are distinct from arsenite induced stress granules (in fact many would argue the new granules are not stress granules on this basis).

We thank the reviewer for these suggestions and have provided further discussion on the presence/absence of eIFs, see line 212.

© 2022. Published by The Company of Biologists under the terms of the Creative Commons Attribution License (https://creativecommons.org/licenses/by/4.0/).

5.In Figure 7A B, the authors measure the impact of FCV on puromycin incorporation. They should also address whether initiation is specifically affected by assessing polysome run-off.

We are currently investigating further the nature of the translation shut-off and the mechanisms responsible for that, as part of follow-on work. We also note that the editor highlighted that no further experimental work was required.

6.In Figure 7C, the authors address potential signaling alterations that correlate with granule formation. The increase in eIF2alpha P is very minimal and requires statistical support if the authors are going to interpret as they currently do. The most convincing change to my mind is the increase in ERK phosphorylation. My understanding is that such an increase is often associated with serum stimulation and increased translation. So this experiment raises many intriguing questions that could easily be addressed by expanding the antibodies used and or the use of some inhibitors.

So does S6 phosphorylation increase? The authors state it doesn't - but I can't find the data here? What about the S6 kinases are these activated? Past literature claims there are connections between stress granules and different S6 kinases (e.g. Eisinger-Mathason et al., 2008. Mol Cell; Sfakianos et al., 2018. Cell Death Diff.). Other past literature connects mild stress with stress granule formation and ERK signaling (e.g. Meyerowitz et al., 2011. Mol Neurodegeneration). So could they use inhibitors of JNK and ERK? With some minimal extra work here, the authors may be able to provide a more concrete signalling mechanism for the formation of these granules.

We have rephrased this section of the results for added clarity, see line 369 and have added data following the reviewer's suggestion. We now show that pharmacological inhibition of ERK1/2 does not impair PG assembly, suggesting ERK1/2 may rather be involved in the downstream effect of PG assembly or other VFS-induced cell changes (Fig. S7C and line 379). In addition, we tested the importance of eIF2a phosphorylation by assessing the impact of ARS or VFS treatment in MEFs expressing WT or non-phosphorylatable S51A eIF2a mutant (Fig. S7A and B and line 372). As previously shown, ARS-induced SG assembly is dependent on eIF2a phosphorylation. In contrast, PG assembly was only reduced to 58% in cells expressing S51A eIF2a, therefore while eIF2a phosphorylation plays a role in PG assembly, it is not essential. Further experiments are underway to investigate a putative role for JNK signalling.

7.The last sentence in the results states 'These suggests that the VFS-induced reduction in viral replication may be due to translational stalling rather than activation of antiviral signalling in cells assembling PGs'. My understanding is that the authors haven't done any experiments to address translational stalling- so this statement needs to be toned down- unless the polysome experiment suggested above gives evidence for translation stalling.
This has been reworded to highlight translation shut-off rather than stalling, see line 397.

Manuscript presentation comments:
1.In Figure 1F, the G3BP1 panels are very faint and difficult to see- the authors should consider replacing this panel with another image or increasing the brightness/contrast so that the G3BP1 signal is more evident.

This has been changed as suggested in the new Fig. 1F.

2.For the time lapse microscopy and other images across the paper- Because the granules following VFS treatment are smaller than the stress granules formed after arsenite treatment, they are more difficult to see. A zoomed in panel for many of the images would help to overcome this problem.

We believe overall that we encountered a conversion issue when trying to accommodate our figures into the overall size requirement for JCS. All images have been re-processed to ensure they are at 300 DPI and panel size increased when possible for clarity.

3.The order of the paper seemed odd in places – as alluded to above the FRAP data may be better with the investigation of G3BP1 requirement. Plus where the FRAP data is presented in Figure 1H- a quantitative assessment of the differences observed in dynamics from the time course might be more interesting.

© 2022. Published by The Company of Biologists under the terms of the Creative Commons Attribution License (https://creativecommons.org/licenses/by/4.0/).

As detailed above the order of the manuscript has been reworked so that the FRAP data leads into the G3BP1 requirement and all FRAP data merged into Fig. S1 for consistency.

4.The final figure deals with the question most general readers are probably asking from the outset- that is does the process where these granules are induced in cells that neighbour infected cells alter their capacity for infection. I would move this to after Fig 1. – so the paper flow would be – 1. The granules form and appear different to stress granules. 2. They are part of a protection mechanism. 3. Their composition and regulation

We have restructured our manuscript as highlighted above, reflecting the constructive comments made here but have kept the protection part last as all authors strongly felt that the manuscript read best when ending on this finding.

Reviewer 3 Comments for the Author:

Major Concerns to be addressed:

1. A very interesting feature of the data that is not highlighted enough by the authors is that UV-inactivated virus (Fig 1A) does not induce PGs yet virus-free supernatant (VFS) does (Fig 1C). It is difficult to understand why this is. At minimum, the authors should provide more information as to the difference between these preparations. For example, UV is used to inactivate any viral particles that remain in the VFS after PEG-precipitation – presumably prior to adding this inactivation step, there was some residual infectivity? Could the authors perform RT-qPCR to quantify viral genomic RNA prior to UV treatment in VFS compared to the viral preps used in Fig 1A? This would quantify the number of inactivated viral particles that are found in each preparation, and help determine if any of the effects observed result from virus particle entry.

We respectfully disagree with this reviewer's assessment of the UV-inactivated results, from his narrative we think this may be due to a misunderstanding and will provide further clarification below.

UV-inactivated viruses are commonly used as control for live virus infection to dissect the impact of viral replication on cellular processes. UV-inactivated viruses have the ability to bind target cells, however they do not replicate and do not yield intracellular viral replication intermediate products or viral proteins. They are a better control than uninfected conditions given they account for receptor binding effect. Therefore, the virus-free supernatant generated from CRFK cells infected with UV-inactivated has been subjected to the same experimental procedures as that from cells infected with live virus, but because UV-inactivated viruses do not replicate one would not expect the cells to respond and secrete the PG inducer as in the case of a live virus. Therefore, it was expected for us that we would not detect PGs in these control conditions. In addition to this, and supported by the point below, we carefully ensured the removal of infectious particle in VFS by both precipitating them and added a further UV crosslinking of the supernatant. Perhaps this double mention of UV-inactivation is what had somehow misled the reviewer in his assessment of the results, and we hope this is now clearer.

Moreover, could the authors try other strategies to better characterize the VFS? DNase? RNase? Proteases treatments? Transwell assays? This could help identify the nature of the molecule(s) that induces the PGs, a central question for any paracrine effect.

In short, this work is ongoing to identify the nature of the PG inducer within VFS from infected cell. We have performed some of the experiment named above and a secretome analysis currently under validation, which will form the basis of a further manuscript.

As the premise of this work is to link these novel granules to IFN or antiviral responses, could the authors examine if the granules form in response to treatment with TypeI IFNs? Or could there be another way to induce PGs that does not rely on a supernatant of an unidentified nature? Perhaps induction of cytoplasmic crowding and LLPS, or overexpression of PG-unique proteins, or a combination of these approaches?

© 2022. Published by The Company of Biologists under the terms of the Creative Commons Attribution License (https://creativecommons.org/licenses/by/4.0/).

This is one of the very first thing we investigated and have data supporting that CRFK cells stimulation with feline IFN does not induce G3BP1 foci induction, see rebuttal figure 1. However, because our manuscript is already figure heavy and at limit permitted by JCS we have decided not to include these data in the final version. We however hope these do satisfy the reviewer’s query.

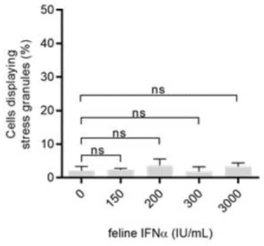

Rebuttal Figure 1: Detection of stress granules following IFN treatment. CRFK cells were treated with increasing concentrations of feline IFNα for 1h, followed by fixation and the distribution of G3BP1 (as SG marker) analysed by immunofluorescence. Bar plot (n=3) of the percentage of cells displaying G3BP1 foci, mean ± SEM for 100 cells analysed across at least 10 acquisitions. Statistical significance shown above the bars, ns: not significant.

Finally, it is important to show the TCID50 assays that were used to verify the absence of infectious viral particles from the VFS and are referred to as data not shown. The preparation and the nature of the VFS is important to understand as it the treatment upon which all subsequent experiments are based.

Similarly to the previous point on the figure and supplementary figure number, we have decided not to include these data in the final version. We however hope that the data presented in the rebuttal figure 2 do satisfy the reviewer’s query.

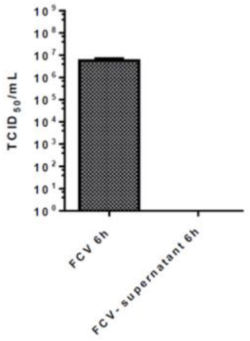

Rebuttal figure 2: Detection of FCV replication by $TCID_{50}$ assay following VFS treatment. CRFK cells were incubated with VFS for 6h, viral titre was then determined by $TCID_{50}$ assay. Results show viral titre from VFS-treated cells compared to the viral titre from untreated FCV-infected cells.

2. A central concern with the study is the circular argument regarding G3BP1. The identification of PGs and most of the analysis shown in this manuscript relied on overexpression of gfp-G3BP1 or immunofluorescence for endogenous G3BP1. The isolation of PGs for extensive proteomic and RNA analysis also relied on overexpressed of gfp-G3BP1, and the pulldown was performed using a gfp antibody, therefore, all the subsequent analysis of the protein and RNA components of PGs has been performed on granules that contain G3BP1. Fig5 shows data that is intended to validate the formation of PGs using antibodies to proteins that are common to PGs and SGs (Fig 5A) and unique to PGs (Fig 5B). In Fig 5B, the panels indicate that cytoplasmic puncta form that are THRAP3 and RBMX positive after VFS treatment and these puncta co-stain with GFP-positive puncta in cells that are overexpressing gfp-G3BP1. There are no cytoplasmic puncta that stain positive for PG-unique marker proteins THRAP3 and RBMX in any cells that do not overexpress gfp-G3BP1. From the data shown, I can only infer that G3BP1 is a required scaffold or catalyst for PG formation. The third panel intended to analyze the PG-unique protein hnRNPK is impossible to decipher because the immunofluorescent staining is of poor quality.

© 2022. Published by The Company of Biologists under the terms of the Creative Commons Attribution License (https://creativecommons.org/licenses/by/4.0/).

These data become more concerning when placed in context with the data shown in Fig S6, wherein the authors state that PGs form in the absence of G3BP1. In these images, cytoplasmic puncta are present in VFS-treated cells in the absence of G3BP1 but these are visualized by staining for FXR1 and UBAP2L – proteins that the authors state are common to PGs and SGs. Therefore, at no time in the manuscript do the authors validate the formation of PGs with PG-specific protein markers like THRAP3 and RBMX in the absence of G3BP1. So that makes me wonder – what did they isolate? Is the granule that is characterized the same granule that is visualized?

I find the inconsistency with these data troubling. Hopefully the authors have additional data that is not yet in the manuscript that can be added to show that PGs form in response to VFS and in the absence of G3BP1 and that these granules can be stained with markers that are unique to PGs, as identified by the authors.

Overall while we disagree with the reviewer's scepticism of our strategy and struggle with the idea that there is inconsistency in the data given the strong support from reviewers #1 and #2 to our approach and results, we provide below more rationale supporting it.

The issue seems to be the use of G3BP1 as a bait for the PG isolation, and the identity of foci isolated. Our approach is based on an established and proven method we and others have applied to characterise SG or SG-like G3BP1 containing foci (Brocard *et al* 2020, Jain *et al* 2016). We appreciate that G3BP1 may not be essential for PG assembly given the data we present, yet it is a component of PG as we show and therefore can be suitable for their isolation. In addition, a criticism is raised regarding which foci are isolated. We are assuming the reviewers posits that PG and perhaps SG may coexist in our VFS treated cells leading to some heterogeneity in the G3BP1-containing foci isolated. However, our CHX treatment experiment clearly show that all foci are resistant to CHX - unlike SGs that are dissolved - and therefore provides reinsurance that only PGs are found in VFS-treated cells. Therefore overall, we are confident about the suitability of our approach.

As for the validation of the MS data, there results are provided in the Supplementary Figure 3. Unlike what the reviewer suggests, the panels are showing that for THRAP3 and RBMX and hnRNPK, when cells are stimulated with VFS both G3BP1 and these markers colocalise, while they remain out from SGs following ARS treatment. To clarify any confusion these cells are now indicated with white arrows.

Minor Concerns:

1.The major premise of the work is that PGs may be a subtype of SGs that are antiviral. The authors do not highlight any IFN signaling molecules found in these granules, and inflammatory cytokines are not altered in cells treated with VFS. In the final figure of the paper, the authors show that VFS pre-treatment limits viral replication, but no evidence is provided to show that the mechanism of this inhibition is anything other than translation shutoff. PGs, although enriched in signaling molecules/their transcripts, do not appear to function in antiviral signaling. This means that PGs do not have a novel function. Combined with the above concerns, I guess I am unclear how different they are from SGs.
To briefly summarize the key differences between PG and SG:

- their protein contents are different (in particular the recruitment of eIFs and ribosomal proteins).
- their requirement for G3BP1 during assembly is different.
- the kinetics of their disassembly is different.
- their sensitivity to cycloheximide treatment is different.

2. I do not understand the disconnect between the more rapid assembly/disassembly properties of PGs relative to SGs (Fig S1) and the observations that FRAP was indistinguishable between PGs and SGs. Is this because G3BP was overexpressed for FRAP making the granule artificially stable and limiting exchange of contents? Please comment in the manuscript on this issue.

While we do show no difference in G3BP1 mobility we cannot exclude that other PG resident proteins, essential to their assembly have different physical properties. It is also possible that PG

© 2022. Published by The Company of Biologists under the terms of the Creative Commons Attribution License (https://creativecommons.org/licenses/by/4.0/).

and SG disassembly may be driven by different pathways (granulophagy, PTMs, specific chaperones) therefore impacting their recovery process as recently shown (Dormann D *et al* Science 2021).

3. Several typos found.

We wholeheartedly apologize for these numerous errors and have corrected all the below in addition to careful proof-reading and editing of further typos.

-please add line numbers to better allow reference to the typos in the text such as:

Done

-PKR listed twice as a signalling molecule recruited to SGs, both in the intro and in results sections

We kept this statement as it reinforces the messages about the link between SGs and innate signalling.

-‘in response in response’ – repeated phrase end of p9

Corrected.

-typo in results re Fig 1E - significant instead of significantly

Corrected.

- assembly-disassembling end of p4

Corrected.

- potential instead of potentially top of p5

Corrected.

- m in mitochondrial is cut off in Fig S5A

Corrected.

- typo in FigS6 legend – SGs should read RLBs I think
Corrected.

Second decision letter

MS ID#: JOCES/2021/259194

MS TITLE: Novel stress granules-like structures are induced via a paracrine mechanism during viral infection.

AUTHORS: Valentina Iadevaia, James M Burke, Lucy Eke, Carla Moller-Levet, Roy Robert Parker, and Nicolas Locker
ARTICLE TYPE: Research Article

We have now reached a decision on the above manuscript.

To see the reviewers' reports and a copy of this decision letter, please go to: https://submit-jcs.biologists.org and click on the 'Manuscripts with Decisions' queue in the Author Area. (Corresponding author only has access to reviews.)

© 2022. Published by The Company of Biologists under the terms of the Creative Commons Attribution License (https://creativecommons.org/licenses/by/4.0/).

As you will see, the reviewers recognize that many of their initial criticisms have been addressed in your revised manuscript. However, reviewers #1 and 3 still raised issues that will require amendments to your manuscript. I hope that you will be able to carry these out, because I would like to be able to accept your paper.

We are aware that you may be experiencing disruption to the normal running of your lab that makes experimental revisions challenging. If it would be helpful, we encourage you to contact us to discuss your revision in greater detail. Please send us a point-by-point response indicating where you are able to address concerns raised (either experimentally or by changes to the text) and where you will not be able to do so within the normal timeframe of a revision. We will then provide further guidance. Please also note that we are happy to extend revision timeframes as necessary.

Please ensure that you clearly highlight all changes made in the revised manuscript. Please avoid using 'Tracked changes' in Word files as these are lost in PDF conversion.

I should be grateful if you would also provide a point-by-point response detailing how you have dealt with the points raised by the reviewers in the 'Response to Reviewers' box. Please attend to all of the reviewers' comments. If you do not agree with any of their criticisms or suggestions please explain clearly why this is so.

<u>Reviewer 1</u>

Advance summary and potential significance to field

Overall, the authors have sufficiently addressed my comments, including new data added with UBAP2L KO cells. I have a few additional points for the new version of manuscript most of which are minor.

Comments for the author

Original comment # 10 – if other blot replicates show similar total eIF2a across three conditions as stated in the rebuttal, one of them should be used to replace this current one, even though it would not be from the same sample.
Otherwise, readers will have this question - why eIF2a becomes downregulated in VFS treated cells.
Fig. 1 – fluorescent channels should be labelled/indicated in color in A,F, and DAPI should be added alongside G3BP1 to C and G.
Fig. 3C – statistical significance (asterisks) indicated in the legend are missing on the panel Fig. S3C. I understand that staining was done for endogenous hnRNPK – yet only two cells contain strong hnRNPK signal in the nucleus in the ARS panel. What happened to nuclear hnRNPK in the adjacent cells? Looks like there is also a large hnRNPK+ granule in one of these two cells in this panel ARS panel.
RBMX was present in the original SG core data set from Jain et al., could authors explain why it was chosen as a PG-specific protein?
Fig. S7B – Is the difference in the number of granules between WT and S51A statistically significant? There is no indication of significance levels but it does look significantly different not only for ARS but also for VFS.
Fig. S5E – could authors comment on RPL9 upregulation in VPS treated cells?

Minor Abstract: triggered by infection – typo
Line 128 – fixed and labelled with an anti-G3BP1 antibody
Line 134 – significantly
Line 178: 'as proposed before' citation required Fig.2C - μM should be changed to μm.
Line 194: How does the absence of a halo indicates that there are pre-existing connections between SG components?
Line 209: what is '946 fold p-value'?
Line 222: punctate should be puncta Line 250: please revise 'GO screening …' sentence as it sounds awkward (also KEGG pathways are not the part of the GO term system).
Line 273: samples

© 2022. Published by The Company of Biologists under the terms of the Creative Commons Attribution License (https://creativecommons.org/licenses/by/4.0/).

Line 274: protein coding
Line 275: consider revising – 'remaining enriched'? What is ‘non processed RNAs’?
Line 300: analysis of PG not SG transcriptome?
Line 388: the conclusion on eIF2a is repeated twice.
Line 396: neat?
Fig.6A – no scale bar
Line 962: remove #21 Please make sure that abbreviations are used consistently throughout the text in particular, stress granule and SG; arsenite and ARS.

Reviewer 2

Advance summary and potential significance to field

The manuscript expands on an exciting observation where following infection with FCV, SGs are induced in the neighboring non-infected cells. The authors reproduce this result using the media collected from infected cells, and show that pre-treatment with this media protects the cells against subsequent challenge with virus. In order to understand these observations, the authors undertake a careful and comprehensive characterization of the granules in terms of composition and dynamics of formation. They also start to dissect some of the signalling pathways that may be involved. As stated in my previous review, I found the paper intriguing and novel.

Comments for the author

The authors have addressed my previous concerns

Reviewer 3

Advance summary and potential significance to field

This is an important study that identifies a new flavour of stress granule that forms in bystander cells in response to virus infection. The study provides a comprehensive analysis of the protein and RNA content of these granules.

The authors have provided good responses to most of the reviewers’ concerns and the manuscript is improved as a result. They have corrected writing/typos and reorganized the data presentation as recommended. The authors have responded to some but not all of my previous concerns, as noted in detail below.

Comments for the author

Major Concern One of my previous major concerns was that the authors did not adequately differentiate their VFS preparation from preparations of UV-inactivated virus. These concerns were not fully addressed in the revised manuscript.
i) The preps of VFS were subjected to PEG precipitation followed by UV inactivation to remove all infectious virus. The authors show data in the response to reviewers to confirm that VFS does not contain infectious virus. I think this one small graph should be added to Fig 1 to validate VFS preps are virus-free.
ii) This reviewer can only assume that the reason the UV inactivation step was added to the VFS preparation conditions is because there remains residual infectious virus after the PEG precipitation that necessitates the additional step of UV-inactivation. This is the reason for my second concern, which was not fully appreciated by the authors. If VFS preps after PEG precipitation do retain virus particles (which I am assuming they do because of the UV inactivation step) then the final VFS preps contain virus particles capable of entry and uncoating even though they are incapable of full replication (and not infectious). The authors do not address the consequence of adding preps that contain low amounts of virus particles onto cells. The control for this is treating with an equal number of UV-inactivated virus particles. The authors do show data in Figure 1 that UV-inactivated FCV does not induce PGs, but the authors do not quantify the amount of virus particles in this UV inactivated prep. This should be done, and equal virus particles should be used as in the VFS

© 2022. Published by The Company of Biologists under the terms of the Creative Commons Attribution License (https://creativecommons.org/licenses/by/4.0/).

treatment. Given the differences in these preparations, I presume that the amount of virus particulate material between these preps is several logs different, making this control inadequate. I would like the authors to show data (perhaps using RT-qPCR for viral RNA in each prep) to quantify the amount of viral genome equivalents are being added in each case. If they are not approximately equal, then the authors should repeat the UV-inactivated control compared to VFS with equivalent virus particles. This is an important control that is currently lacking from Figure 1 of the paper.
iii) The premise of the paper is that there is a paracrine effect – a molecule or molecules released from infected cells induces PGs in bystander cells. Have the authors examined if FCV infection of multiple cell types produces VFS that is capable of induction of PGs? Do all cell types express the molecule(s) after infection that induces this PG effect?
Minor Concerns Fig 1G – are PGs increased in size or number in response to VFS + CHX treatment? Authors should comment on this or change the representative image which looks like PGs are bigger and more numerous with both treatments.
Fig 2A – what are the UBAP2L-positive granules that form after arsenite treatment in U20S G3BP1/2 KO cells? Please comment.
Fig 6C – As stated by the authors, there is clearly no increase in p38 signaling but there appears to be a decrease in total and phosphor-p38 relative to the mock control – the authors should note this if it is a consistent observation. Would inhibition of p38 signaling increase PG formation? It would be interesting to test the effect of p38 manipulation with chemical inhibitors and add these data to Fig S7.
Line 397 – it is incorrect to say that the dilution of VFS rescued viral replication. Rather, the diluted VFS failed to prevent viral replication. Please reword.

Second revision

Author response to reviewers' comments

Dear Dr. Maria Carmo-Fonseca,

Thank you for your initial assessment of our study entitled ‘Novel stress granules-like structures are induced via a paracrine mechanism during viral infection’ (JOCES/2021/259194) and for giving us the opportunity to revise our manuscript.

As you suggested, we have revised our manuscript and provided clarifications/corrections where requested.

We do hope that you will find this improved version of our manuscript suitable for publication in Journal of Cell Science.

Kind regards
Nicolas Locker

Response to Reviewer #1:
We fully appreciate the comments made by this reviewer and took them onboard to improve our manuscript as outlined in our response to their comments.

Original comment # 10 – if other blot replicates show similar total eIF2a across three conditions as stated in the rebuttal, one of them should be used to replace this current one, even though it would not be from the same sample. Otherwise, readers will have this question - why eIF2a becomes downregulated in VFS treated cells.
This has now been amended as suggested in the new Figure 6B.

Fig. 1 – fluorescent channels should be labelled/indicated in color in A,F, and DAPI should be added alongside G3BP1 to C and G.
done.

© 2022. Published by The Company of Biologists under the terms of the Creative Commons Attribution License (https://creativecommons.org/licenses/by/4.0/).

Fig. 3C – statistical significance (asterisks) indicated in the legend are missing on the panel
We are not sure Fig 3C nor its legend have a reference to asterisks so have left this unchanged.

Fig. S3C. I understand that staining was done for endogenous hnRNPK – yet only two cells contain strong hnRNPK signal in the nucleus in the ARS panel. What happened to nuclear hnRNPK in the adjacent cells? Looks like there is also a large hnRNPK+ granule in one of these two cells in this panel ARS panel. RBMX was present in the original SG core data set from Jain et al., could authors explain why it was chosen as a PG-specific protein?
As noted by this reviewer we found the hnRNPK signal to vary from cell-to-cell with some displaying very weak hnRNPK signal throughout the selected fields of view. We are unclear whether this is a feature of the particular antibody used or a property of U2OS cells. As for the isolated large granules hnRNPK+, we note this is an isolated granule (one out of >25 on this panel) and likely not biologically relevant in the context of our analysis.
In our hands, RBMX did not concentrate in SG following arsenite stimulation (this was also the case following thapsigargin treatment – data not shown), while it accumulated in PG following VFS treatment and thus was chosen as an example of PG-specific resident protein.

Fig. S7B – Is the difference in the number of granules between WT and S51A statistically significant? There is no indication of significance levels but it does look significantly different not only for ARS but also for VFS.
We have added statistical analysis to the figure and legends as suggested.

Fig. S5E – could authors comment on RPL9 upregulation in VPS treated cells?
We are currently exploring further the regulation of ribosomal protein mRNAs following VFS treatment. These are known to contain 5′-TOP (5′-terminal oligopyrimidine tract) elements important for their expression, and are under the control of mTOR signalling pathway, but have also been found to be regulated by TIA-1/TIAR proteins which are resident SG proteins (PMID: 21979918). We are following up on this lead to understand whether SG/PG proteins play a role in ribosomal protein expression given our observation in Figure 6 that the Akt/mTOR axis did not seem to be triggered by VFS treatment.

Minor
Abstract: triggered by infection – typo
done.

Line 128 – fixed and labelled with an anti-G3BP1 antibody
done.

Line 134 – significantly
done.

Line 178: ‘as proposed before’ citation required
done.

Fig.2C - μM should be changed to μm.
done.

Line 194: How does the absence of a halo indicates that there are pre-existing connections between SG components?
To be clearer there is a halo present which reflects the background binding to beads of immature SG/PGs (or large G3BP1 RNPs), as discussed in the text.

Line 209: what is ‘946 fold p-value’?
Corrected in the text, (946 fold enrichment, p-value…).

Line 222: punctate should be puncta
done.

Line 250: please revise ‘GO screening …’ sentence as it sounds awkward (also KEGG pathways are not the part of the GO term system).

© 2022. Published by The Company of Biologists under the terms of the Creative Commons Attribution License (https://creativecommons.org/licenses/by/4.0/).

done.

Line 273: samples
done.

Line 274: protein coding
done.

Line 275: consider revising – 'remaining enriched'? What is 'non processed RNAs'?
corrected.

Line 300: analysis of PG not SG transcriptome?
corrected.

Line 388: the conclusion on eIF2a is repeated twice.
corrected.

Line 396: neat?
Corrected to undiluted for clarity.

Fig.6A – no scale bar
Scale bar added.

Line 962: remove #21
done.

Please make sure that abbreviations are used consistently throughout the text, in particular, stress granule and SG; arsenite and ARS.
done.

Response to Reviewer #3:

We fully appreciate the comments made by this reviewer, some of which we feel had already been addressed in our previous rebuttal and revised manuscript but took them onboard and provide further explanations as outlined in our response below.

Major Concern

One of my previous major concerns was that the authors did not adequately differentiate their VFS preparation from preparations of UV-inactivated virus. These concerns were not fully addressed in the revised manuscript.

i) The preps of VFS were subjected to PEG precipitation followed by UV inactivation to remove all infectious virus. The authors show data in the response to reviewers to confirm that VFS does not contain infectious virus. I think this one small graph should be added to Fig 1 to validate VFS preps are virus-free.

Done. To satisfy this query, we have added this data to Figure 1 in the panel highlighting the preparation of the VFS.

ii) This reviewer can only assume that the reason the UV inactivation step was added to the VFS preparation conditions is because there remains residual infectious virus after the PEG precipitation that necessitates the additional step of UV-inactivation. This is the reason for my second concern, which was not fully appreciated by the authors. If VFS preps after PEG precipitation do retain virus particles (which I am assuming they do because of the UV inactivation step) then the final VFS preps contain virus particles capable of entry and uncoating even though they are incapable of full replication (and not infectious). The authors do not address the consequence of adding preps that contain low amounts of virus particles onto cells. The control for this is treating with an equal number of UV-inactivated virus particles. The authors do show data in Figure 1 that UV-inactivated

© 2022. Published by The Company of Biologists under the terms of the Creative Commons Attribution License (https://creativecommons.org/licenses/by/4.0/).

FCV does not induce PGs, but the authors do not quantify the amount of virus particles in this UV inactivated prep. This should be done, and equal virus particles should be used as in the VFS treatment. Given the differences in these preparations, I presume that the amount of virus particulate material between these preps is several logs different, making this control inadequate. I would like the authors to show data (perhaps using RT-qPCR for viral RNA in each prep) to quantify the amount of viral genome equivalents are being added in each case. If they are not approximately equal, then the authors should repeat the UV-inactivated control compared to VFS with equivalent virus particles. This is an important control that is currently lacking from Figure 1 of the paper.

We still do not fully understand where the reviewer's concerns lay and why there is a major concern. However, we have tried below to provide further explanation and rationale for our approach.

Based on our observations that seemingly uninfected cells assemble SG-like puncta during FCV infection, we posited that these may result from the release of a messenger molecule from infected cells inducing paracrine signalling and assembly of these puncta in bystander cells. To test this hypothesis, we had to collect the cell supernatants from infected cells in culture and deplete viral particles which normally egress from infected cells during the infection process. To this end we adapted standard protocols for viral particles isolation (the PEG/NaCl precipitation and centrifugation) and added a further UV inactivation step to ensure that no viral material could be left after this procedure. This was then further checked by infection assays as discussed in our previous rebuttal and now shown in the new Figure 1. UV-inactivated particles are precipitated and removed by the centrifugation step – and this is in fact how FCV stocks are prepared routinely – therefore we disagree that the VFS preparation does contain viral particles. We also disagree with this reviewer that we should attempt to measure this by further means –'I would like the authors to show data (perhaps using RT-qPCR for viral RNA in each prep) to quantify the amount of viral genome equivalents are being added in each case'. As discussed in our previous rebuttal, we are currently investigating the nature of the paracrine messenger using proteomics analysis of VFS preparation, and while this does constitute a separate study, we can share this analysis has identified peptides of feline origin and potential messenger hits that we are currently investigating, while we could not detect any peptide of viral origin. This adds further confidence that viral particles – inactivated or not - are not present in the VFS preparation. In addition, attempts to identify the biochemical nature of the messenger led us to test various VFS inactivation procedures and the data shown in Rebuttal Figure 1 highlights that VFS is insensitive to RNAse addition, which would degrade viral RNA from viral particles for example. Again, this supports that VFS preparations are virus-free.

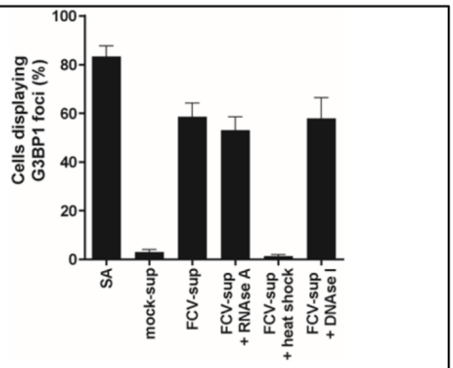

Rebuttal Figure 1: PG assembly is not affected by DNAse or RNAse treatment. VFS was first treated with either RNAse A for 30mn, DNAse I for 30mn at 37C or heated for 20mn at 65C, before being added to CRFK cells for 1h to induce PG assembly. PG assembly, or SG assembly following sodium arsenite (SA) treatment, was then assessed by confocal microscopy. Samples were stained for the SG marker G3BP1 and the percentage of CRFK cells displaying G3BP1 foci, mean ± SD for 100 G3BP1-positive cells analysed across at least 10 acquisitions.

We hope this now satisfies the reviewer's query.

© 2022. Published by The Company of Biologists under the terms of the Creative Commons Attribution License (https://creativecommons.org/licenses/by/4.0/).

iii) The premise of the paper is that there is a paracrine effect – a molecule or molecules released from infected cells induces PGs in bystander cells. Have the authors examined if FCV infection of multiple cell types produces VFS that is capable of induction of PGs? Do all cell types express the molecule(s) after infection that induces this PG effect?

This is an interesting comment. Unfortunately, the cell line models available to study FCV infection (a feline virus) are mainly restricted to CRFK cells, with only a handful of papers referring at Feline embryonic fibroblasts (FEA) cells as infectible. We do not currently have access to these cell lines. Future and on-going work in our laboratory will aim at exploring whether VFS generated from other virus models (-ssRNA, +dsRNA or DNA viruses) also result in the assembly of PGs.

Minor Concerns

Fig 1G – are PGs increased in size or number in response to VFS + CHX treatment? Authors should comment on this or change the representative image which looks like PGs are bigger and more numerous with both treatments.
Comment added in the text.

Fig 2A – what are the UBAP2L-positive granules that form after arsenite treatment in U20S G3BP1/2 KO cells? Please comment.
This is exactly what has been described by Cirillo and co-workers in PMID: 31956030, where they discovered that UBAP2L forms distinct cores that act in nucleating stress granules upstream of G3BP1. In this study, they proposed a model in which UBAP2L is an essential SG nucleator that acts upstream of G3BP1/2 forming small RNPs, and facilitates G3BP1 core formation and SG assembly and growth. These are the same foci we observed, consistent with this earlier study.

Fig 6C – As stated by the authors, there is clearly no increase in p38 signaling but there appears to be a decrease in total and phosphor-p38 relative to the mock control – the authors should note this if it is a consistent observation. Would inhibition of p38 signaling increase PG formation? It would be interesting to test the effect of p38 manipulation with chemical inhibitors and add these data to Fig S7.
Comment on this observation has been added as suggested. We will take onboard the suggestion to follow up p38 inhibition for example using SB203580 as we previously did in the context of DENV or MNV infection.

Line 397 – it is incorrect to say that the dilution of VFS rescued viral replication. Rather, the diluted VFS failed to prevent viral replication. Please reword.
Reworded as suggested.

<u>Third decision letter</u>

MS ID#: JOCES/2021/259194

MS TITLE: Novel stress granules-like structures are induced via a paracrine mechanism during viral infection.

AUTHORS: Valentina Iadevaia, James M Burke, Lucy Eke, Carla Moller-Levet, Roy Robert Parker, and Nicolas Locker
ARTICLE TYPE: Research Article

I am happy to tell you that your manuscript has been accepted for publication in Journal of Cell Science, pending standard ethics checks.

© 2022. Published by The Company of Biologists under the terms of the Creative Commons Attribution License (https://creativecommons.org/licenses/by/4.0/).
